# African swine fever virus MGF505-4R facilitates cGAS degradation through TOLLIP-mediated selective autophagy and inhibits the formation of ISGF3 to evade innate immunity

**DOI:** 10.1186/s13567-025-01569-x

**Published:** 2025-07-05

**Authors:** Manman Yao, Pengfei Li, Xiangmin Li, Wentao Li, Ping Qian

**Affiliations:** 1 National Key Laboratory of Agricultural Microbiology, Hubei Hongshan Laboratory, Huazhong Agricultural University, Wuhan, 430070 Hubei China; 2 College of Veterinary Medicine, Huazhong Agricultural University, Wuhan, 430070 Hubei China; 3 Key Laboratory of Preventive Veterinary Medicine in Hubei Province, The Cooperative Innovation Center for Sustainable Pig Production, Wuhan, 430070 Hubei China

**Keywords:** ASFV, MGF505-4R, degradation, autolysosome, cGAS-STING, immune evasion, JAK-STAT, STAT1, STAT2, type I interferon

## Abstract

African swine fever virus (ASFV) causes acute and highly lethal disease in pigs. To counteract host defense systems and facilitate virus infection, many ASFV-encoded proteins have regulatory effects on the innate immune response. In this study, we constructed an MGF505-4R-deleted ASFV strain (ASFV-Δ4R) and found that, compared with the wild-type ASFV, ASFV-Δ4R infection significantly increased IFN-β production and elevated the mRNA levels of antiviral genes in porcine alveolar macrophages. Mechanistically, MGF505-4R interacts with cGAS and promotes its degradation by triggering Toll-interacting protein (TOLLIP)-mediated selective autophagy. Specifically, MGF505-4R enhanced the interaction between cGAS and TOLLIP, which subsequently led to increased degradation of cGAS. Additionally, MGF505-4R inhibited IFN-β-induced signal transmission by interacting with STAT1 and STAT2 and impeding their phosphorylation. This effect consequently prevented the formation of the ISGF3 heterotrimer and its subsequent translocation to the nucleus, leading to the downregulation of antiviral genes. As expected, compared with the ASFV-WT strain, ASFV-Δ4R infection increased phosphorylation of STAT1 and STAT2 and subsequent ISGF3 formation, leading to an elevated expression of antiviral gene ISGs. This discovery enhances the understanding of the immune regulation strategies evolved by ASFV and offers valuable perspectives for antiviral research targeting ASFV.

**Supplementary Information:**

The online version contains supplementary material available at 10.1186/s13567-025-01569-x.

## Introduction

African swine fever (ASF) is a highly infectious and devastating disease that impacts the global pig industry. Symptoms caused by highly virulent strains include hemorrhaging, high fever, and diarrhea, resulting in a high mortality rate approaching 100% [1, 2]. The African swine fever virus (ASFV) is the causative agent of ASF. It is the sole recognized DNA arbovirus within the genus *Asfivirus*, family *Asfarviridae* [3]. ASFV is a double-stranded DNA (dsDNA) virus with a large genome encoding more than 150 proteins. These proteins play critical roles in various processes, including viral assembly, replication, virulence, and immune evasion. However, many of these viral proteins remain poorly characterized. Owing to the existing knowledge gap regarding the architecture of the giant dsDNA virus, the clarification of its complex protein components, and the identification of sophisticated immune manipulation strategies, no commercial vaccine has been successfully developed for the control of ASFV [4–6].

As a cytosolic DNA sensor, cyclic GMP-AMP synthase (cGAS) catalyzes the production of 2′3′-cyclic GMP-AMP (2′3′-cGAMP) in response to the invasion of DNA viruses. This molecule subsequently engages with stimulator of interferon genes (STING), which then recruits TANK-binding kinase 1 (TBK1) to phosphorylate interferon regulatory factor 3 (IRF3), thereby initiating type I interferon (IFN-I) responses [7–9]. IFN-I then activates the downstream signaling cascade by attaching to its receptor subunits, IFNAR1 and IFNAR2. Following this, Janus kinase 1 (JAK1) and tyrosine kinase 2 (TYK2) are activated, which then phosphorylate signal transducer and activator of transcription 1 (STAT1) and STAT2. These proteins subsequently associate with interferon regulatory factor 9 (IRF9) to generate interferon-stimulated gene factor 3 (ISGF3). This compound enters the nucleus and attaches to the interferon-stimulated regulatory element (ISRE), triggering the expression of various interferon-stimulated genes (ISGs) and establishing an antiviral state [10, 11].

Multigene families (MGFs) are located in the terminal regions of the genome and are classified into several distinct categories on the basis of their average protein length [12, 13]. Many of these proteins are implicated in viral virulence and evasion of the innate immune response, thereby influencing viral pathogenicity. Studies have indicated that the combined deletion of the MGF360 and MGF505 genes leads to decreased virulence and growth impairments [14, 15]. Another study revealed that MGF-360-10 L mediates JAK1 degradation by engaging the E3 ubiquitin ligase HERC5. The pathogenicity of ASFV CN/GS/2018 significantly decreases when MGF-360-10 L is removed [16]. Additionally, MGF-505-7R has been demonstrated to interact with STING. Compared with that of the wild-type strain, the replicative capacity of MGF505-Δ7R is notably diminished [17]. Similarly, MGF505-3R disrupts the cGAS-STING signaling pathway through targeting TBK1 for degradation [18]. Furthermore, MGF-505-7R interacts with JAK1 and JAK2, leading to their degradation [19].

Macroautophagy, commonly referred to as autophagy, is a fundamental and dynamic catabolic process that captures macromolecules inside autophagosomes, which subsequently merge with lysosomes for degradation [20]. While autophagy was originally perceived as a self-digestion process, it is now widely recognized that autophagy can selectively degrade cytosolic components through various autophagy receptors, including neighbor of BRCA1 (NBR1), sequestosome 1 (SQSTM1/p62), calcium binding and coiled-coil domain 2 (CALCOCO2/NDP52), optineurin (OPTN), and Toll-interacting protein (TOLLIP). These receptors bind to specific cargoes, such as intracellular pathogens, misfolded proteins, or aberrant organelles, subsequently directing them to the phagophores for breakdown via their LC3-interacting regions (LIRs) [21, 22].

In this study, we discovered that ASFV-Δ4R induced stronger antiviral gene expression than its parental strain. Mechanistically, we found that ASFV MGF505-4R antagonizes the cGAS‒STING axis through interactions with cGAS. Specifically, MGF505-4R was shown to downregulate cGAS protein level by recruiting the autophagy receptor TOLLIP to initiate selective autophagy, thereby promoting cGAS degradation. In addition, MGF505-4R was found to impair the JAK-STAT axis through its interaction with STAT1 and STAT2. Mechanistically, MGF505-4R inhibited the phosphorylation of both STAT1 and STAT2, which in turn hindered the assembly of the ISGF3 complex and its movement into the nucleus. This ultimately led to the downregulation of ISG expression. Compared with the parental strain, the ASFV-Δ4R strain partially lost its ability to inhibit interferon beta (IFN-β)-induced phosphorylation of STAT1 and STAT2 and subsequent ISG expression. These findings reveal a novel mechanism by which MGF505-4R regulates the cGAS‒STING and JAK‒STAT signaling pathways, enhancing our understanding of ASFV immune evasion and offering insights for antiviral research.

## Materials and methods

### Biosafety statement and facility

All experiments involving active African swine fever virus (ASFV) were conducted in a Biosafety Level 3 (BSL-3) facility at Huazhong Agricultural University (Wuhan, China), with approval from the Ministry of Agriculture and Rural Affairs of the People’s Republic of China.

### Cells and viruses

HEK293T (ATCC, CRL-11268), HeLa (ATCC, CCL-2), PK-15 (ATCC, CCL-33), LLC-PK1 (ATCC, CL-101), and swine kidney-6 (SK6; Wuhan University) cells were maintained in DMEM (HyClone, SH30022.01) supplemented with 10% FBS (Gibco, 16000044) and 1% penicillin‒streptomycin (GENVIEW, GA3502). Porcine alveolar macrophages (PAMs) were isolated from 20 to 30-day-old piglets and cultured in RPMI 1640 medium (Gibco, 11875119), as previously described [23]. ASFV SXH1 was isolated following established protocols [23], and GFP-PRV was propagated and stored at −80 °C as described earlier [24].

### Plasmids

The full-length MGF505-4R gene was cloned into the pTRIP-CMV vector (Addgene, 102611) with a 6 × His tag. Plasmids encoding cGAS, STING, TBK1, IKKϵ, and IRF3-5D were constructed and stored in our laboratory as previously described [25]. Flag-tagged STAT1, STAT2, and IRF9 were generated using the pTRIP-CMV vector. Additionally, NBR1, p62, NDP52, OPTN, and TOLLIP were cloned into the pcDNA3.1-HA vector (Addgene, 128034). For dual luciferase assays, pRL-TK, pGL3-IFN-β-Luc, and pGL3-ISRE-Luc plasmids were used as described previously [25].

### Reagents and antibodies

The mouse anti-MGF505-4R antibody was kindly provided by Han Jun (China Agricultural University). The mouse anti-p72 antibody was prepared as previously described [23]. The following mouse monoclonal antibodies were obtained from Proteintech: His-tag (66005-1-Ig), JAK1 (66466-1-Ig), GAPDH (60004-1-Ig), TYK2 (67411-1-Ig), and HA-tag (66006-2-Ig). Additionally, rabbit polyclonal antibodies against cGAS (26416-1-AP), STING (19851-1-AP), IRF3 (11312-1-AP), phospho-IRF3 (29528-1-AP), STAT1 (10144-2-AP), STAT2 (16674-1-AP), IRF9 (14167-1-AP), LC3B (187251-AP), and Flag-tag (20543-1-AP) were procured from the same source. Rabbit polyclonal antibodies targeting phospho-STAT1 (AF3300) and phospho-STAT2 (AF3342) were purchased from Affinity Bioscience. Rabbit His-tag polyclonal antibodies (PM032), mouse Flag-tag monoclonal antibodies (M185-3 L), goat anti-mouse polyclonal antibodies (72-8042-M001), and goat anti-rabbit polyclonal antibodies (72-8067-M001) were purchased from Medical and Biological Laboratories. The 2'-3'-cyclic GMP-AMP (HY-16658B), the caspase inhibitor Z-VAD-FMK (HY-16658B), and the autophagosome inhibitor CQ (HY-17589A) were obtained from MedChemExpress. The proteasome inhibitors MG132 (S1748-1 mg), NH_4_Cl (ST2030-100 g), DMSO (ST038-100 mL), and CHX (66-81-9) were purchased from Sigma‒Aldrich.

### Construction of the MGF505-4R gene deletion ASFV

The ASFV SXH1 strain served as the skeleton to construct a mutant with the deletion of the MGF505-4R gene via homologous recombination. Specifically, the recombinant transfer vector PUC57-p72EGFP-Δ4R was constructed. This vector contains a 1003 bp left homologous arm positioned before MGF505-4R, an EGFP reporter gene regulated by the p72 promoter, and a 1012 bp right homologous arm situated after MGF505-4R. Afterward, the PUC57-p72EGFP-Δ4R transfer vector was transfected into LLC-PK1 cells for 24 h before infection with ASFV SXH1. The virus was harvested 48 h post-infection and subsequently used to infect PAMs until increasing EGFP expression was observed. The MGF505-4R-deficient mutant was purified through successive limiting dilutions and plaque purification in PAMs. The deletion of MGF505-4R in the recombinant virus was confirmed via specific primers that target both the terminal and central regions of the MGF505-4R gene. All primers used for ASFV-WT and ASFV-Δ4R detection are listed in (Additional file 1).

### RT‒qPCR

Total RNA extraction, reverse transcription, and qPCR were performed as previously described [26]. Briefly, the treated cells were lysed using TRIpure reagent (Aidlab Technologies, RN0102) for RNA extraction. cDNA was synthesized using a reverse transcription kit (Yeasen, 11142ES10), followed by qPCR with SYBR Green Master Mix (YEASEN, 11202ES03). Data were analyzed using the 2-ΔΔCT method, and all qPCR primers are listed in (Additional file 2).

### Dual-luciferase reporter assay

The cells were transfected with reporter plasmids pGL3-IFN-β-Luc or pGL3-ISRE-Luc, along with pRL-TK and other relevant plasmids. After 24 h of transfection, the cells were treated with the specified inducer and analyzed using a Reporter Gene Assay Kit (Beyotime Biotechnology, RG028).

### Western blot and co-IP analysis

The cells were lysed in NP-40 lysis buffer (Beyotime Biotechnology, P0013F), and a portion of the supernatant was detected by 12% SDS‒PAGE, followed by transfer to a PVDF membrane (Roche, 46978100) for western blot analysis. For co-IP assays, the remaining lysates were incubated with specific antibodies for 4 h, followed by the addition of 30 μL Protein A + G agarose (Beyotime Biotechnology, P2055-2 mL) and a 2 h incubation. The samples were then washed five times with NP-40 lysis buffer to remove nonspecific binding before proceeding with western blot analysis as described above.

### Nuclear and cytoplasmic extraction

HeLa cells were transfected with increasing doses of His-MGF505-4R plasmids for 24 h, followed by treatment with IFN-β (Proteintech, HZ-1298) for 2 h. Afterward, the cells were isolated via a Nuclear and Cytoplasmic Protein Extraction Kit (Beyotime Biotechnology, P0027).

### RNA interference

To downregulate the expression of cGAS, ATG5, and TOLLIP, small interfering RNAs (siRNAs) targeting cGAS (sicGAS), ATG5 (siATG5), and TOLLIP (siTOLLIP) were synthesized by Sangon Biotech (Shanghai, China). The siRNA was transfected into HeLa cells via Lipofectamine 3000 (Thermo Fisher Scientific, L3000150) for 24 h. All siRNA sequences are included in (Additional file 3).

### Immunofluorescence assay

HEK293T cells grown on coverslips were transfected with relevant plasmids for 24 h, after which they were fixed with 4% paraformaldehyde and permeabilized with 0.1% Triton X-100 (Beyotime Biotechnology, P0096) for 10 min. Subsequently, the cells were blocked with 2% bovine serum albumin before being incubated with the indicated antibodies. The nuclei were stained with 4’,6-diamidino-2-phenylindole (Invitrogen, 62247). Finally, the coverslips were fixed onto slides and observed under a confocal laser scanning microscope.

### Enzyme-linked immunosorbent assay

To detect the secreted IFN-β from the treated cells, the supernatants from the transfected HeLa cells and PAMs infected with Mock, ASFV-WT, or ASFV-Δ4R were collected. These samples were then analyzed via IFN-β ELISA kits following the manufacturer’s guidelines.

### Statistical analysis

The data were processed using GraphPad Prism 8.0 and presented as means ± SD from at least two independent experiments. Statistical significance was determined by an unpaired, two-tailed Student’s *t*-test assuming unequal variances (*p < 0.05; **p < 0.01; ***p < 0.001; ns, not significant).

## Results

### Compared with ASFV-WT, ASFV-Δ4R induces stronger IFN-I responses

**Figure 1:**
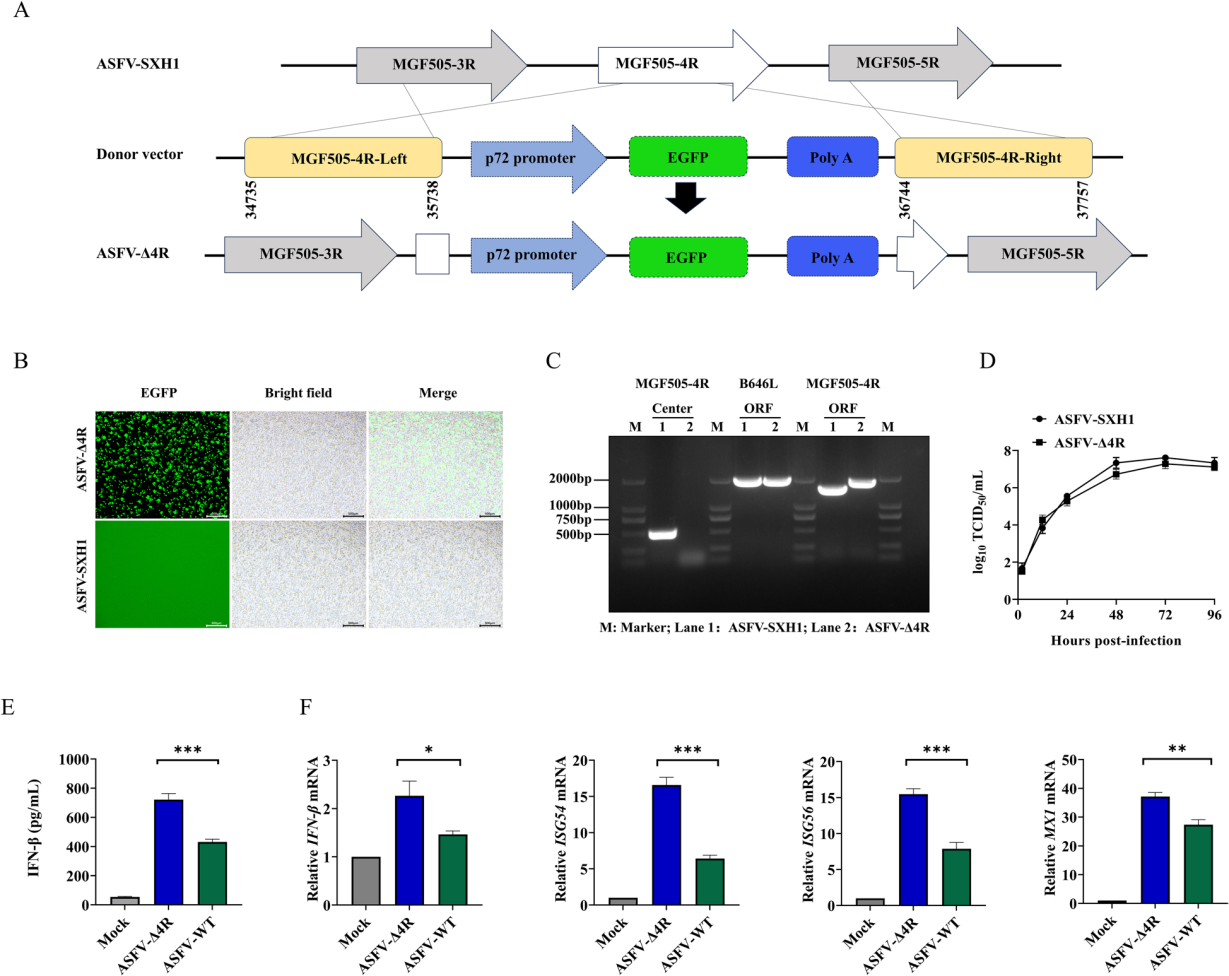
**Compared with ASFV-WT, ASFV-Δ4R induces enhanced IFN-I responses**. **A** MGF505-4R-deleted mutant (ASFV-Δ4R) was constructed from the parental strain of ASFV SXH1 via homologous recombination, as illustrated in the schematic diagram. **B** The purification of ASFV-Δ4R was achieved through a series of successive limiting dilutions, followed by a screening process based on the fluorescence of EGFP in PAMs. **C** PCR analyses of the ASFV-WT and purified ASFV-Δ4R strains via primers that target the central and ORF regions of MGF505-4R. **D** PAMs were infected with 1 MOI of ASFV-WT or ASFV-Δ4R, and the viral titers were measured at the indicated time points. **E** PAMs were either mock-infected or infected with ASFV-WT or ASFV-Δ4R for 36 h before IFN-β secretion was measured via ELISA kits. **F** PAMs were either mock-infected or infected with ASFV-WT or ASFV-Δ4R for 36 h. The mRNA levels of IFN-β, ISG54, ISG56, and MX1 were detected via RT‒qPCR. *n* = 3; *p < 0.05; **p < 0.01; ***p < 0.001; ns, not significant.

Our previous study identified ASFV MGF505-4R as a potent inhibitor of Sendai virus-induced IFN-β promoter activation among 29 ASFV MGF members [26]. In this study, PRV-GFP was used to test whether MGF505-4R inhibits dsDNA virus-induced IFN-I responses. The dual-luciferase assay revealed that MGF505-4R overexpression inhibited the activation of the IFN-β promoter induced by PRV-GFP, thereby increasing PRV-GFP replication in both HEK293T and SK6 cells (Additional file 4). To verify the opposing function of MGF505-4R in the IFN-I response during ASFV infection, ASFV SXH1, a previously isolated genotype II strain from clinical samples [23], was used to construct an ASFV-Δ4R mutant by deleting the MGF505-4R gene through homologous recombination, as shown in Figure 1A. The recombinant virus was subjected to a series of limiting dilutions and fluorescence screenings (Figure 1B). PCR assays revealed that no corresponding band was detected in the ASFV-Δ4R strain when primers targeting the central segment of the MGF505-4R gene were used. However, a band for the reporter gene cassette was observed in the ASFV-Δ4R strain when using primers that target the terminal regions of MGF505-4R (Figure 1C), suggesting that the ASFV-Δ4R strain was successfully constructed and purified. Interestingly, ASFV-WT and ASFV-Δ4R presented similar growth kinetics at all time points, suggesting that the removal of the MGF505-4R gene had no significant effect on the replication of ASFV in PAMs (Figure 1D). To investigate the role of MGF505-4R in ASFV-mediated immune regulation, we subsequently tested the secreted IFN-β in the supernatants of PAMs infected with Mock, ASFV-WT, or ASFV-Δ4R via ELISA kits and found that, compared with the ASFV-WT strain, ASFV-Δ4R induced higher levels of secreted IFN-β (Figure 1E). Furthermore, the mRNA levels of IFN-β, ISG54, ISG56, and MXI induced by ASFV-Δ4R were significantly higher than those in PAMs infected with ASFV-WT (Figure 1F). These data demonstrate that MGF505-4R is involved in dampening IFN-I responses during ASFV infection.

### ASFV MGF505-4R targets cGAS to intercept the cGAS‒STING signaling pathway

**Figure 2:**
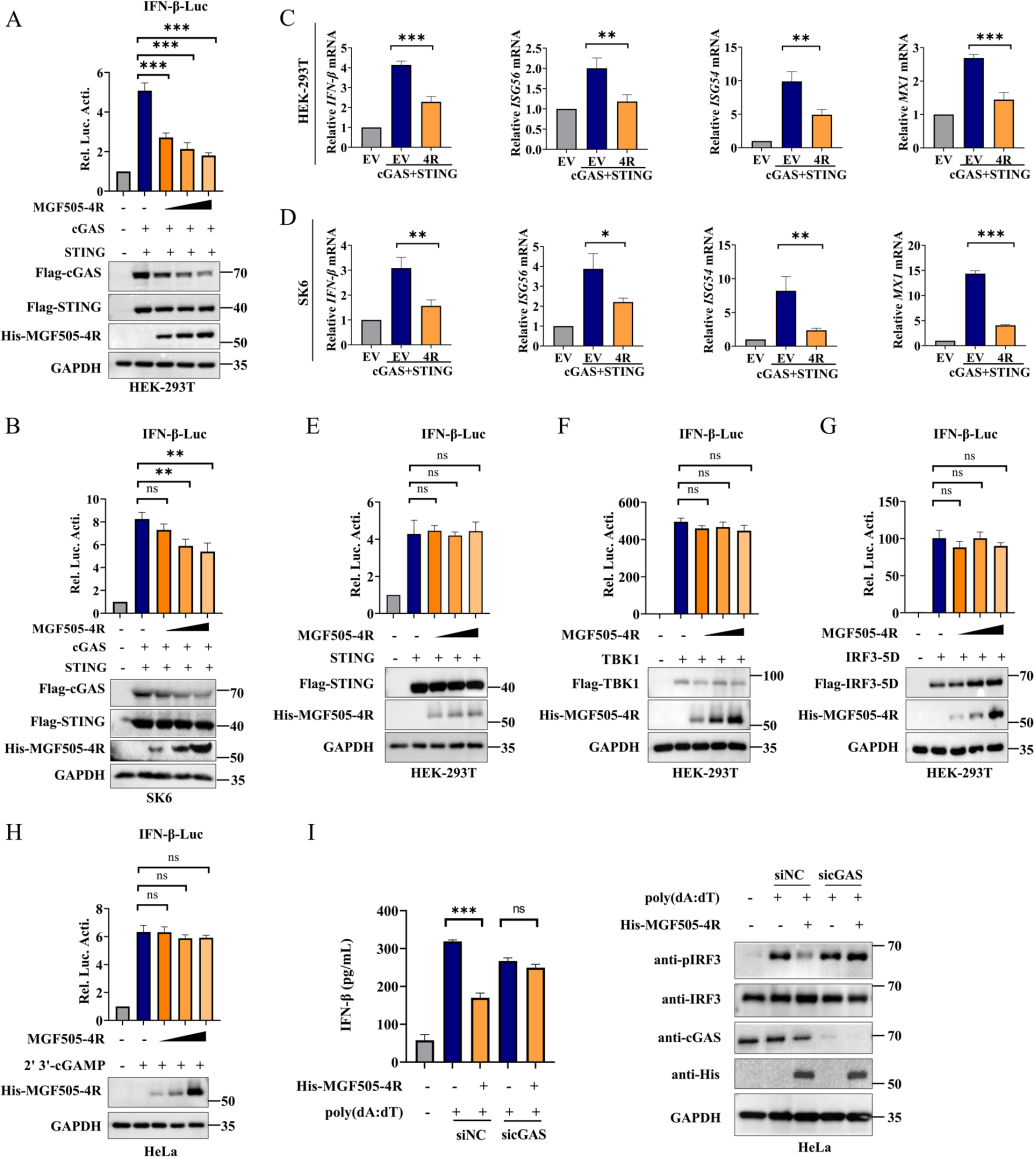
**ASFV MGF505-4R targets cGAS to intercept the cGAS‒STING signaling pathway.** **A** and **B** HEK293T (**A**) and SK6 (**B**) cells were transfected with pGL3-IFN-β-Luc (0.1 μg), pRL-TK (0.05 μg), Flag-cGAS, and Flag-STING, along with increasing doses of His-MGF505-4R plasmids, for 24 h before luciferase and western blot assays. **C** and **D** HEK293T (**C**) and SK6 (**D**) cells were transfected with Flag-cGAS, Flag-STING, or increasing doses of His-MGF505-4R plasmids for 24 h before the detection of IFN-β, ISG54, ISG56, and MX1 mRNA levels. **E–G** HEK293T cells were transfected with Flag-STING (**E**), Flag-TBK1 (**F**), or Flag-IRF3-5D (**G**), along with pGL3-IFN-β-Luc, pRL-TK and increasing doses of His-MGF505-4R plasmids, for 24 h before luciferase assays. **H** HeLa cells were transfected with the His-MGF505-4R plasmid along with pGL3-IFN-β-Luc and pRL-TK for 24 h. Subsequently, the cells were treated with 2′3’-cGAMP for an additional 12 h before luciferase assays. **I** HeLa cells were transfected with negative control siRNA or siRNA targeting cGAS, along with His-MGF505-4R plasmids, for 24 h. Subsequently, the cells were retransfected with poly(dA:dT) for 12 h. The IFN-β levels in the supernatants of treated cells were measured via ELISA kits, and the protein levels were detected using western blot analysis. *n* = 3; *p < 0.05; **p < 0.01; ***p < 0.001; ns, not significant.

Previous research has indicated that MGF505-4R likely targets TRAF3 to inhibit the RIG-I-MAVS signaling pathway [27]. In this study, we further investigated whether MGF505-4R is involved in regulating the cGAS‒STING axis, a crucial DNA-sensing pathway. The dual-luciferase assay revealed that MGF505-4R significantly suppressed the activation of the IFN-β promoter triggered by cGAS and STING in both HEK293T and SK6 cells (Figures 2A and B). Consistent with these results, MGF505-4R also inhibited the cGAS-STING-induced mRNA expression of IFN-β, ISG54, ISG56, and MX1 in both HEK293T and SK6 cells (Figures 2C and D). To further explore the specific signaling molecules regulated by MGF505-4R, we conducted dual-luciferase assays by coexpressing MGF505-4R with key components of cGAS-STING pathway. The results showed that MGF505-4R had no significant impact on the IFN-β promoter activation induced by STING, TBK1, or IRF3-5D in HEK293T cells, indicating that MGF505-4R primarily targets proteins upstream of STING (Figures 2E–G). Additionally, we found that MGF505-4R had no significant effect on the activation of the IFN-β promoter induced by the second messenger 2′3′-cGAMP in HeLa cells (Figure 2H). To determine whether MGF505-4R targets cGAS, we synthesized three siRNAs targeting cGAS and evaluated their knockdown efficiency in HeLa cells. Among these, siRNA 2# demonstrated the most effective knockdown and was selected for subsequent experiments (Additional file 5A). Next, cGAS-knockdown and wild-type HeLa cells were transfected with MGF505-4R and simulated with poly(dA:dT), following that the IFN-β levels in the supernatant of treated cells were tested using an ELISA kit, the results demonstrated that MGF505-4R failed to inhibit dsDNA-triggered IFN-β production and IRF3 phosphorylation in the cGAS-knockdown cells, but it was effective in wild-type HeLa cells (Figure 2I). These findings collectively indicate that cGAS is a potential target of MGF505-4R in modulating IFN-I responses.

### ASFV MGF505-4R interacts with cGAS

**Figure 3:**
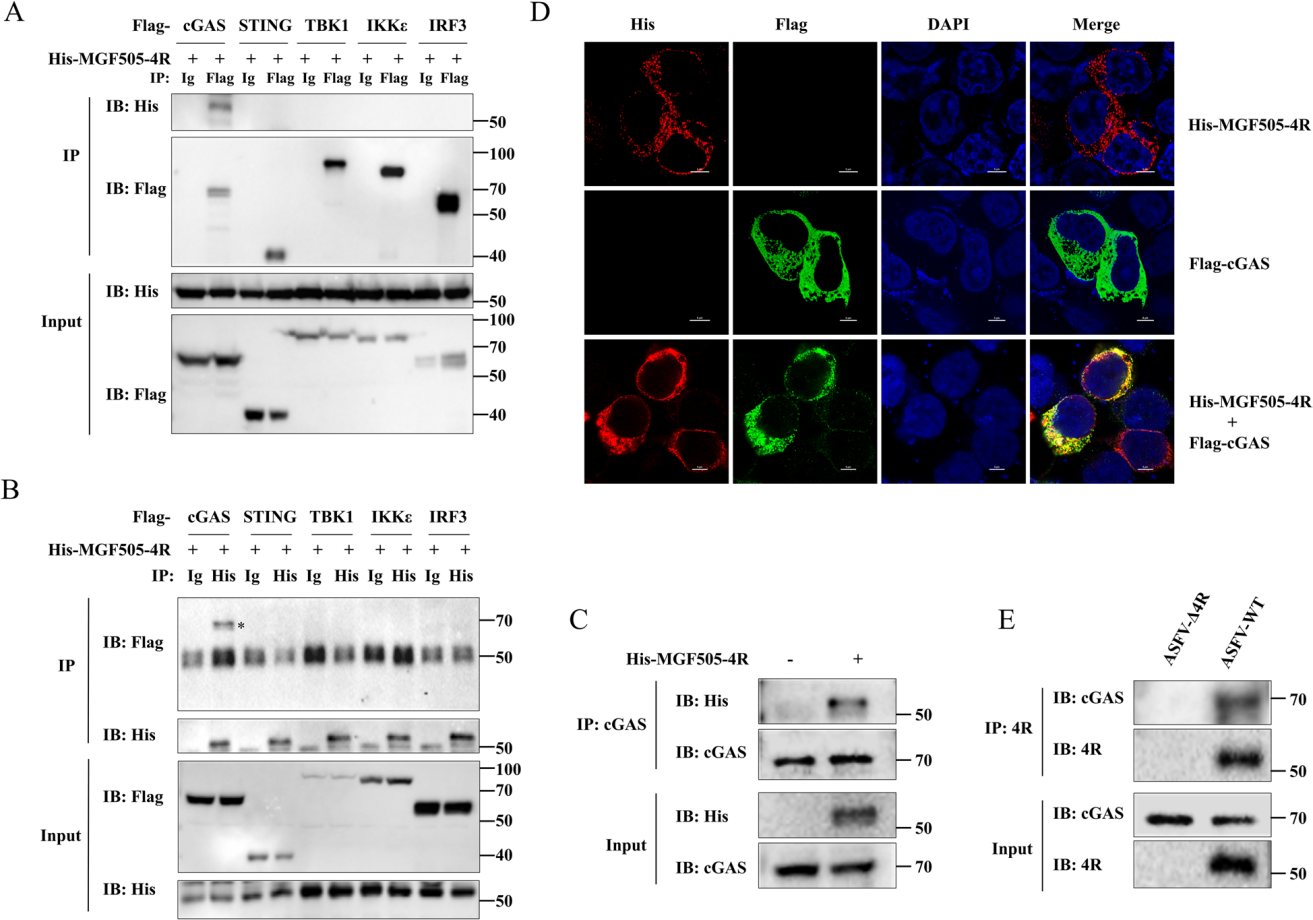
**ASFV MGF505-4R interacts with cGAS.** **A** and **B** HEK293T cells were individually transfected with Flag-cGAS, Flag-STING, Flag-TBK1, Flag-IKKε, or Flag-IRF3-5D, along with His-MGF505-4R plasmids, for 24 h before western blotting and co-IP detection via anti-Flag-labeled (**A**) or anti-His-labeled (**B**) protein A + G agarose. **C** HeLa cells were transfected with the His-MGF505-4R plasmid for 24 h before co-IP detection with anti-cGAS-labeled protein A + G agarose. **D** HEK293T cells were transfected with Flag-cGAS and His-MGF505-4R plasmids for 24 h before confocal microscopy observation. **E** PAMs were infected with ASFV-WT or ASFV-Δ4R for 36 h before western blot and co-IP analysis.

We further investigated whether MGF505-4R interacts with cGAS via coimmunoprecipitation (co-IP). The results indicated that His-MGF505-4R was able to coprecipitate with Flag-cGAS but not with Flag-STING, Flag-TBK1, Flag-IKKϵ, or Flag-IRF3-5D when both anti-Flag- and anti-His-labeled agarose were used (Figures 3A and B). To verify the interactions between endogenous cGAS and MGF505-4R, HeLa cells transfected with the MGF505-4R plasmid were subjected to western blotting and co-IP assays. The results suggested that MGF505-4R coprecipitated with cGAS when using anti-cGAS-conjugated agarose (Figure 3C). Furthermore, colocalization between Flag-cGAS and His-MGF505-4R was observed via confocal microscopy in HEK293T cells (Figure 3D). Importantly, we conducted co-IP analysis using anti-4R-labeled agarose under the condition of ASFV infection, and found that MGF505-4R coprecipitated with cGAS during ASFV-WT infection in PAMs (Figure 3E). Collectively, these data demonstrate that MGF505-4R interacts with cGAS to regulate the cGAS‒STNG axis.

### ASFV MGF505-4R mediates cGAS degradation through the autophagy‒lysosome pathway

**Figure 4:**
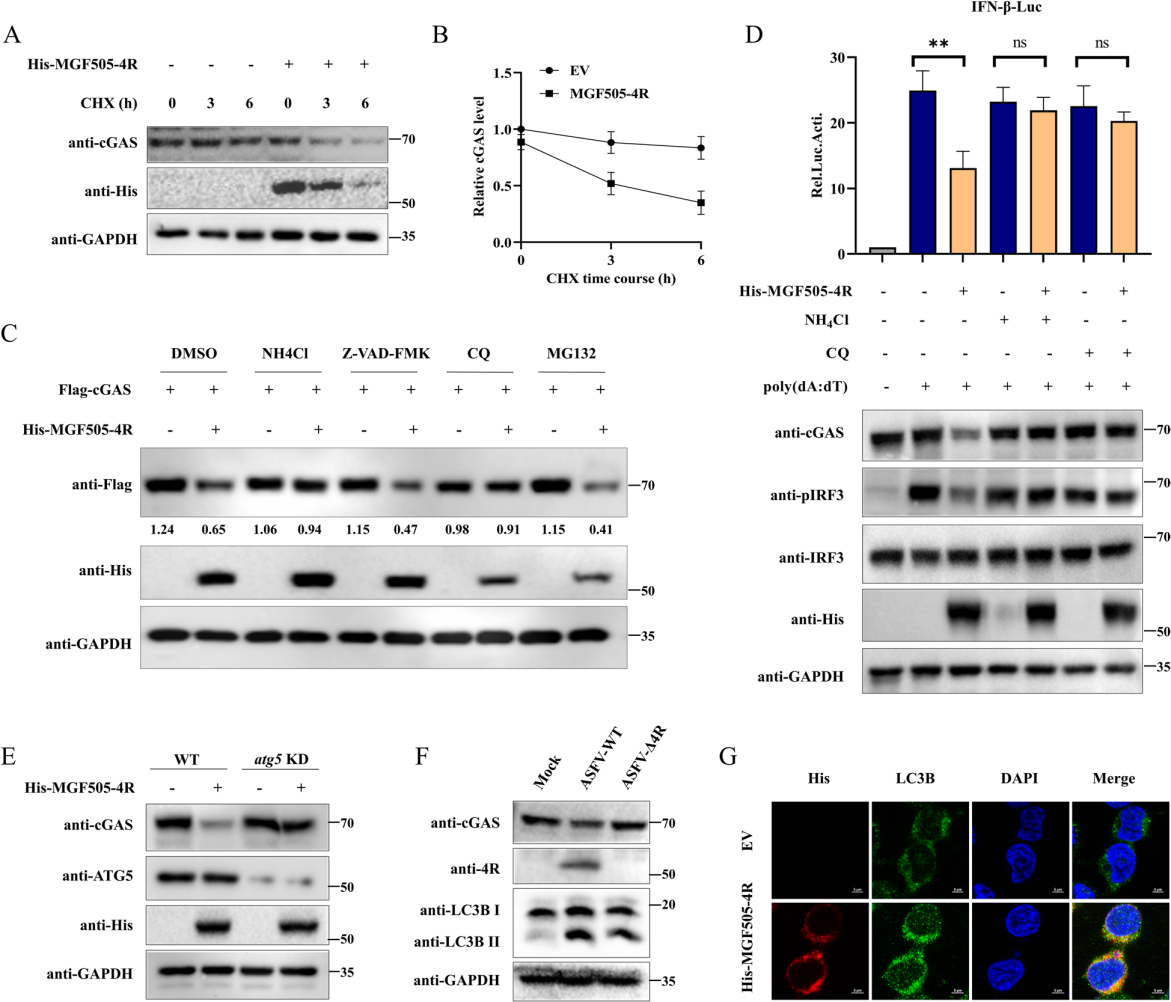
**ASFV MGF505-4R mediates cGAS degradation through the autophagy‒lysosome pathway. A** and **B** HeLa cells were transfected with either an empty vector or His-MGF505-4R plasmids for 24 h and then treated with CHX for 0 h, 3 h, or 6 h before western blot detection (**A**). The relative cGAS protein levels were analyzed via ImageJ (**B**). **C** HEK293T cells were transfected with Flag-cGAS, along with empty vector or His-MGF505-4R plasmids, for 24 h and then treated with DMSO (negative control), NH_4_Cl (20 mM), Z-VAD-FMK (50 μM), CQ (20 μM), or MG132 (10 μM) for 12 h before western blot detection. **D** HeLa cells were transfected with pGL3-IFN-β-Luc and pRL-TK, along with His-MGF505-4R plasmids, for 24 h. Subsequently, the cells were treated with NH_4_Cl or CQ for 12 h and subsequently retransfected with poly(dA:dT) for an additional 12 h before luciferase and western blot assays. **E** HeLa cells were transfected with siRNA targeting ATG5 for 24 h and then retransfected with the His-MGF505-4R plasmid for 24 h before western blot analysis. **F** PAMs were infected with Mock, ASFV-WT, or ASFV-Δ4R for 36 h before western blot analysis. **G** HEK293T cells were transfected with empty vector or His-MGF505-4R plasmids for 24 h before IFA detection with the indicated antibodies. *n* = 3; **p < 0.01; ns, not significant.

Our earlier research revealed that MGF505-4R had a suppressive effect on the protein level of cGAS (Figures 2A, B, and I). To investigated whether MGF505-4R mediates the degradation of cGAS at the protein level, HeLa cells overexpressing MGF505-4R were treated with cycloheximide (CHX) to block protein synthesis, and the cGAS protein levels were detected at various time points. The results demonstrated that MGF505-4R dramatically reduced the cGAS protein level compared to the control group following CHX treatment (Figures 4A and B). Furthermore, we examined the pathway regulated by MGF505-4R in the degradation of the cGAS protein. HEK293T cells overexpressing His-MGF505-4R and Flag-cGAS were separately treated with the protease inhibitor MG132, the autophagy‒lysosome inhibitors NH_4_Cl and chloroquine (CQ), or the caspase inhibitor z-VAD-FMK prior to western blot analysis. The results indicated that NH_4_Cl and CQ, but not MG132 or z-VAD-FMK, rescued MGF505-4R-mediated cGAS degradation (Figure 4C). Consistently, MGF505-4R facilitated the degradation of endogenous cGAS, followed by reduced IFN-β promoter activation and phosphorylation of IRF3 triggered by poly(dA:dT) in HeLa cells. However, this effect was inhibited by treatment with NH_4_Cl and CQ (Figure 4D). To further verify this results, three siRNAs targeting autophagy-related protein 5 (ATG5) were synthesized, and their knockdown effects were tested in HeLa cells. Among these, siRNA 1# showed best effects and was selected for further experiments (Additional file 5B). Next, we transfected MGF505-4R into both ATG5-knockdown and control HeLa cells, and assessed cGAS protein levels using western blot. The data presented that MGF505-4R failed to mediate cGAS degradation in ATG5-knockdown HeLa cells (Figure 4E). On the basis of these data, we further examined the effects of MGF505-4R on autophagy during ASFV infection. Compared with ASFV-Δ4R infection, ASFV-WT infection induced greater conversion of LC3B-I to LC3B-II in PAMs (Figure 4F). Consistently, the ectopic expression of MGF505-4R induced a punctate distribution of LC3B in HEK293T cells (Figure 4G). Taken together, these findings indicate that MGF505-4R facilitates cGAS degradation by triggering autophagy.

### ASFV MGF505-4R facilitates the degradation of cGAS through TOLLIP-mediated selective autophagy

**Figure 5:**
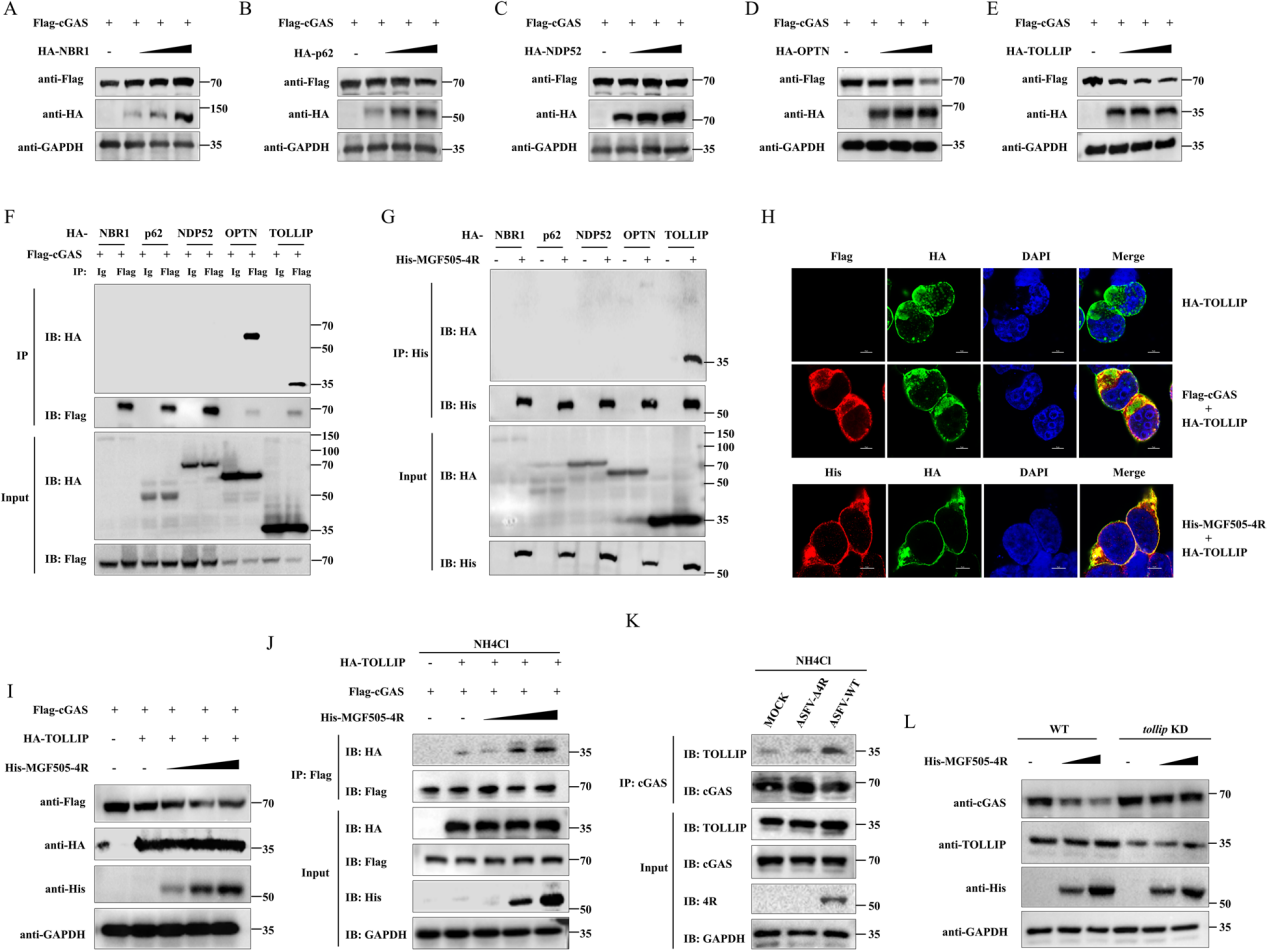
**ASFV MGF505-4R facilitates the degradation of cGAS through TOLLIP-mediated selective autophagy.** **A**–**E** HEK293T cells were transfected with the Flag-cGAS plasmid along with increasing doses of the HA-NBR1 (**A**), HA-p62 (**B**), HA-NDP52 (**C**), HA-OPTN (**D**), or HA-TOLLIP (**E**) plasmid for 24 h before western blot analysis. **F** HEK293T cells were transfected with the Flag-cGAS plasmid along with the HA-NBR1, HA-p62, HA-NDP52, HA-OPTN, or HA-TOLLIP plasmids for 24 h before co-IP and western blot assays. **G** HEK293T cells were co-transfected with His-MGF505-4R plasmid along with HA-NBR1, HA-p62, HA-NDP52, HA-OPTN, or HA-TOLLIP plasmids for 24 h prior to co-IP and western blot assays. **H** HEK293T cells were co-transfected with Flag-cGAS and HA-TOLLIP or His-MGF505-4R and HA-TOLLIP for 24 h and then subjected to IFA detection. **I** HEK293T cells were transfected with Flag-cGAS and HA-TOLLIP, along with increasing doses of His-MGF505-4R plasmids, for 24 h before western blot analysis. **J** HEK293T cells were transfected with Flag-cGAS and HA-TOLLIP, along with increasing doses of His-MGF505-4R plasmids for 24 h. Subsequently, the cells were treated with NH_4_Cl for 12 h before western blot and co-IP assays. **K** PAMs were mock-infected or infected with ASFV-WT or ASFV-Δ4R for 24 h and then treated with NH_4_Cl for 12 h before western blot and co-IP analysis. **L** HeLa cells were transfected with either negative control siRNA or siRNA targeting TOLLIP for 24 h. Subsequently, the cells were retransfected with the His-MGF505-4R plasmid for an additional 24 h before western blot analysis.

To further elucidate the mechanism by which MGF505-4R facilitates the degradation of cGAS, we screened the potential autophagy receptors associated with this process. The plasmids encoding the autophagy receptors (OPTN, TOLLIP, p62, NBR1, and NDP52) were individually co-transfected with plasmid expressing cGAS, and protein levels were analyzed by western blot. The results indicated that OPTN and TOLLIP, but not p62, NBR1 or NDP52, led to a significant reduction in the cGAS protein level (Figures 5A–E). Additionally, we found that cGAS coprecipitated with OPTN and TOLLIP but not with p62, NBR1 or NDP52, using anti-Flag-labeled agarose (Figure 5F). Next, to examine the relationships between MGF505-4R and these autophagy receptors, HEK293 T cells co-expressing His-MGF505-4R along with HA-OPTN, HA-TOLLIP, HA-p62, HA-NBR1, or HA-NDP52 were subjected to co-IP analysis. The results revealed that His-MGF505-4R could interact with TOLLIP but not with the other receptors (Figure 5G). Consistently, colocalization was observed between TOLLIP and cGAS, as well as between TOLLIP and MGF505-4R in IFA (Figure 5H). To further investigate how MGF505-4R influences the interaction between TOLLIP and cGAS, HEK293 T cells were co-transfected with plasmids encoding His-MGF505-4R, HA-TOLLIP, and Flag-cGAS, followed by western blot and co-IP analysis. The results showed that MGF505-4R dose-dependently facilitated TOLLIP-mediated cGAS degradation (Figure 5I). Furthermore, MGF505-4R increased the interaction between TOLLIP and cGAS (Figure 5J). To determine if MGF505-4R regulates the interaction between endogenous cGAS and TOLLIP during ASFV infection, we performed co-IP assay in PAMs infected with ASFV-WT or ASFV-Δ4R. Compared to infection with ASFV-Δ4R, infection with ASFV-WT increased the interaction between endogenous cGAS and TOLLIP in PAMs (Figure. 5K). To investigate whether TOLLIP is required for MGF505-4R-mediated cGAS degradation, three siRNAs targeting TOLLIP were synthesized, and their knockdown effects were tested in HeLa cells. Among these, siRNA 3# showed best effects and was selected for further experiments (Additional file 5C). We next examined cGAS protein levels by western blot in wild-type and TOLLIP-knockdown HeLa cells overexpressing MGF505-4R. As expected, the degradation of endogenous cGAS induced by MGF505-4R was attenuated when TOLLIP was knocked down (Figure 5L). These findings demonstrate that MGF505-4R triggers TOLLIP-mediated selective autophagy to facilitate cGAS degradation.

### MGF505-4R suppresses the IFN-β-mediated signaling pathway

**Figure 6:**
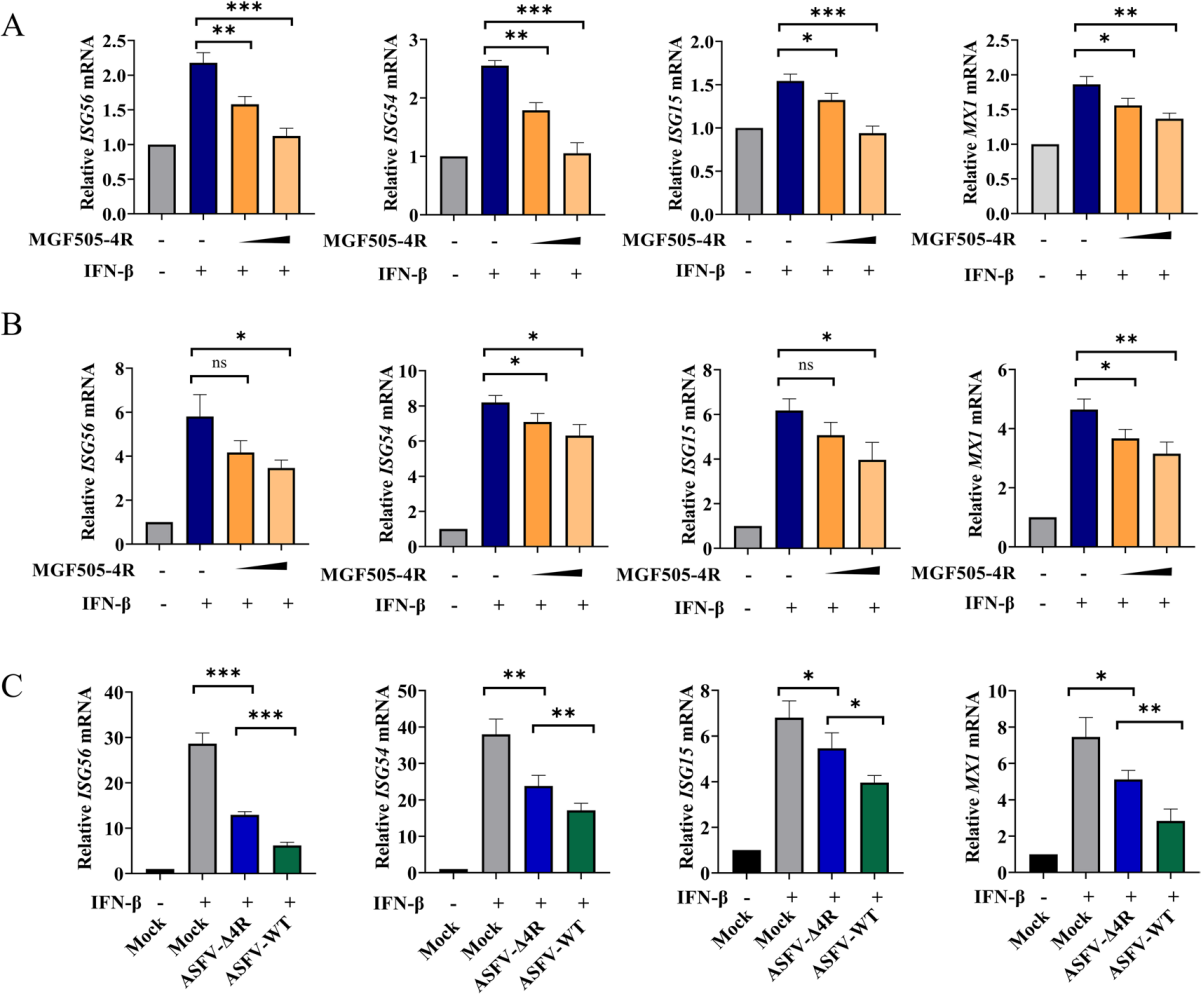
**MGF505-4R suppresses the IFN-β-mediated signaling pathway.** **A** and **B** HEK293T (**A**) and PK15 (**B**) cells were transfected with increasing doses of His-MGF505-4R plasmids for 24 h before the detection of ISG56, ISG54, ISG15, and MX1 mRNA levels. **C** PAMs were either mock-infected or infected with ASFV-WT or ASFV-Δ4R for 36 h and then treated with IFN-β for 2 h before the mRNA levels of ISG56, ISG54, ISG15, and MX1 were measured. *n* = 3; *p < 0.05; **p < 0.01; ***p < 0.001; ns, not significant.

The production of IFN subsequently stimulates the JAK-STAT signaling pathway, leading to the upregulation of numerous ISGs and thereby eliciting a broad range of antiviral responses [28]. To further investigate whether MGF505-4R directly regulates the IFN-β-mediated JAK-STAT signaling pathway, we first tested the mRNA levels of ISGs during MGF505-4R overexpression in the presence of IFN-β treatment. Notably, the results showed that MGF505-4R inhibited IFN-β-induced ISG56, ISG54, ISG15, and MX1 mRNA expression in HEK293T and PK15 cells (Figures 6A and B). To determine whether MGF505-4R regulates IFN-β-induced ISGs expression during ASFV infection, PAMs infected with ASFV-WT or ASFV-Δ4R were subjected to RT-qPCR detection. Consistently, ASFV-WT infection markedly dampened the mRNA levels of ISG54, ISG56, ISG15, and MX1, which are spurred by IFN-β in PAMs. However, compared with infection with ASFV-WT, ASFV-Δ4R infection partially lost these effects on PAMs when the MGF505-4R gene was deleted (Figure 6C). Overall, these data demonstrate that MGF505-4R effectively dampens the IFN-β-mediated pathway.

### ASFV MGF505-4R interacts with STAT1 and STAT2

**Figure 7:**
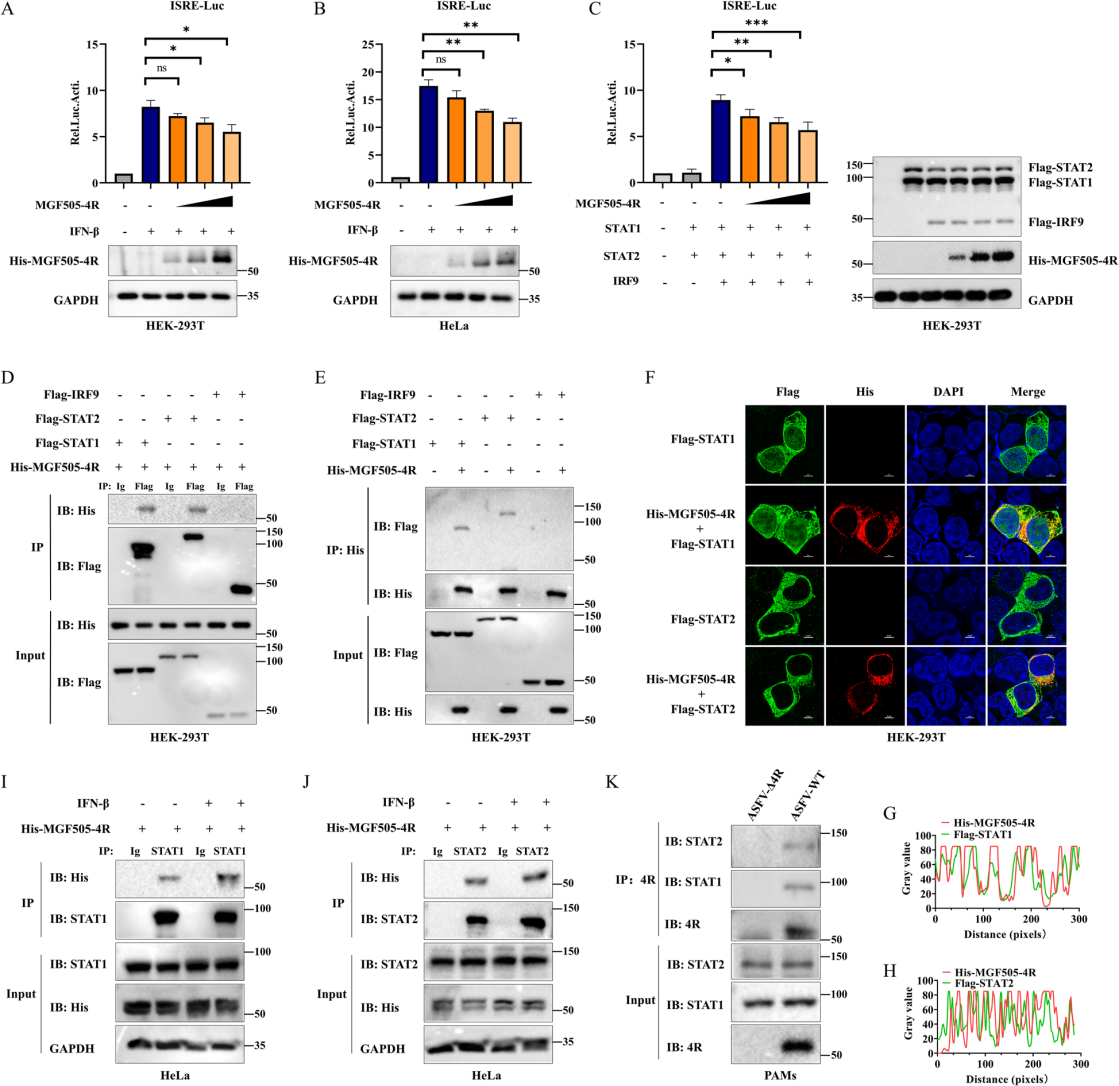
**ASFV MGF505-4R interacts with STAT1 and STAT2.** **A** and **B** HEK293T (**A**) and HeLa (**B**) cells were transfected with pGL3-ISRE-Luc and pRL-TK along with increasing doses of His-MGF505-4R plasmids for 24 h and then treated with IFN-β for 2 h before luciferase and western blot assays. **C** HEK293T cells were transfected with pGL3-ISRE-Luc, pRL-TK, Flag-STAT1, Flag-STAT2, or Flag-IRF9, along with increasing doses of His-MGF505-4R plasmids, for 24 h before luciferase and western blot assays. **D** and **E** HEK293T cells were transfected with His-MGF505-4R along with Flag-STAT1, Flag-STAT2, or Flag-IRF9 for 24 h. Subsequently, the cells were subjected to co-IP assays using anti-Flag-labeled agarose (**D**) and anti-His-labeled agarose (**E**). **F** HEK293T cells were transfected with either empty vector or His-MGF505-4R plasmids, along with Flag-STAT1 or Flag-STAT2 plasmids, for 24 h before IFA was performed with the indicated antibodies. **G** and **H** The colocalization of His-MGF505-4R and Flag-STAT1 (**G**) and that of His-MGF505-4R and Flag-STAT2 (**H**) were analyzed via ImageJ. **I** and **J** HeLa cells were transfected with the His-MGF505-4R plasmid for 24 h and then treated with or without IFN-β for 2 h before co-IP was performed with anti-STAT1 (**I**) and anti-STAT2 (**J**) antibodies. **K** PAMs were infected with ASFV-WT or ASFV-Δ4R for 36 h before western blot and co-IP analysis. *n* = 3; *p < 0.05; **p < 0.01; ***p < 0.001; ns, not significant.

To further investigate how MGF505-4R influences the JAK‒STAT pathway. HEK293T and HeLa cells overexpressing MGF505-4R were treated with IFN-β. Dual luciferase assay revealed that MGF505-4R inhibited the activation of the ISRE promoter induced by IFN-β in HEK293T and HeLa cells (Figures 7A and B). Furthermore, MGF505-4R overexpression dose-dependently suppressed ISGF3-induced ISRE promoter activation in HEK293T cells (Figure 7C). Next, we conducted co-IP experiments to determine whether interactions exist between MGF505-4R and ISGF3. The findings revealed that His-MGF505-4R was associated with Flag-STAT1 and Flag-STAT2 but not with Flag-IRF9 via both anti-Flag-labeled agarose and anti-His-labeled agarose (Figures 7D and E). Consistently, the red fluorescence of His-MGF505-4R significantly overlapped with the green fluorescence of Flag-STAT1 and Flag-STAT2 under a confocal microscope, as analyzed via ImageJ (Figures 7F–H). We further investigated the interactions between His-MGF505-4R and the endogenous proteins STAT1 and STAT2 in HeLa cells. Our findings revealed that His-MGF505-4R notably interacted with STAT1 and STAT2 both in the presence and absence of IFN-β (Figures 7I and J). Additionally, MGF505-4R was able to coprecipitate with STAT1 and STAT2 during infection with ASFV-WT (Figure 7K). These findings demonstrate that MGF505-4R interacts with STAT1 and STAT2 to modulate the JAK‒STAT pathway.

### ASFV MGF505-4R impedes ISGF3 formation

**Figure 8:**
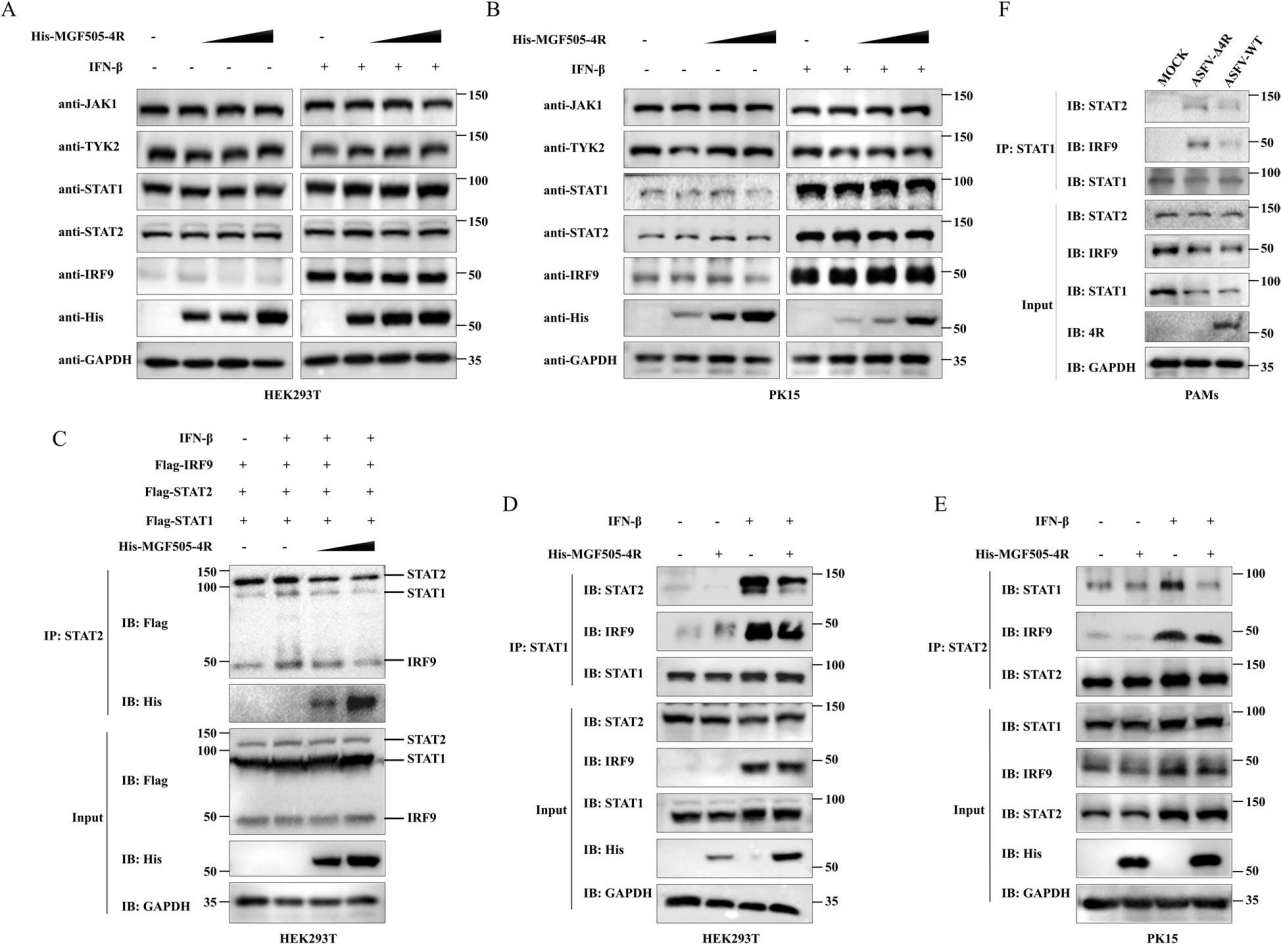
**MGF505-4R impedes ISGF3 formation**. **A** and **B** HEK293T (**A**) and PK15 (**B**) cells were transfected with increasing doses of His-MGF505-4R plasmids for 24 h. Subsequently, the cells were treated with IFN-β for 2 h before western blot analysis. **C** HEK293T cells were transfected with increasing doses of His-MGF505-4R, along with Flag-STAT1, Flag-STAT2, and Flag-IRF9 plasmids, for 24 h and then treated with IFN-β for 2 h before co-IP and western blot assays. **D** and **E** HEK293T (**D**) and PK15 (**E**) cells were transfected with either empty vector or His-MGF505-4R plasmids for 24 h and then treated or untreated with IFN-β for 2 h before co-IP and western blot assays. **F** PAMs were mock-infected or infected with ASFV-WT or ASFV-Δ4R for 36 h before western blot and co-IP analysis.

The regulation of protein levels is a crucial strategy for modulating the activation of signaling pathways. We assessed the protein levels of key components in JAK-STAT signaling pathway in HEK293T and PK15 cells overexpressing MGF505-4R, and found that MGF505-4R had no effect on the endogenous protein levels of JAK1, TYK2, STAT1, STAT2, or IRF9 (Figures 8A and B). The formation of the ISGF3 heterotrimer is a prerequisite to activate the JAK‒STAT pathway. Therefore, we performed co-IP experiments during the overexpression of His-MGF505-4R, Flag-STAT1, Flag-STAT2, and Flag-IRF9. The results indicated that Flag-STAT2 coprecipitated a reduced amount of Flag-STAT1 and Flag-IRF9 during the overexpression of MGF505-4R in HEK293T cells (Figure 8C). To further verify this point, we investigated whether MGF505-4R affects endogenous ISGF3 formation under IFN-β treatment. The results suggested that MGF505-4R overexpression reduced the coprecipitation of STAT1, STAT2, and IRF9 via the use of anti-STAT1-conjugated agarose in HEK293T cells and anti-STAT2-conjugated agarose in PK15 cells (Figures 8D and E). Compared with ASFV-Δ4R infection, ASFV-WT infection consistently decreased the binding of STAT1, STAT2 and IRF9 (Figure 8F). These data demonstrate that MGF505-4R disrupts the interactions among STAT1, STAT2, and IRF9, thereby suppressing the formation of the ISGF3 heterotrimer and the activation of the JAK‒STAT pathway.

### ASFV MGF505-4R prevents the migration of phosphorylated STAT1, phosphorylated STAT2, and IRF9 into the nucleus

**Figure 9:**
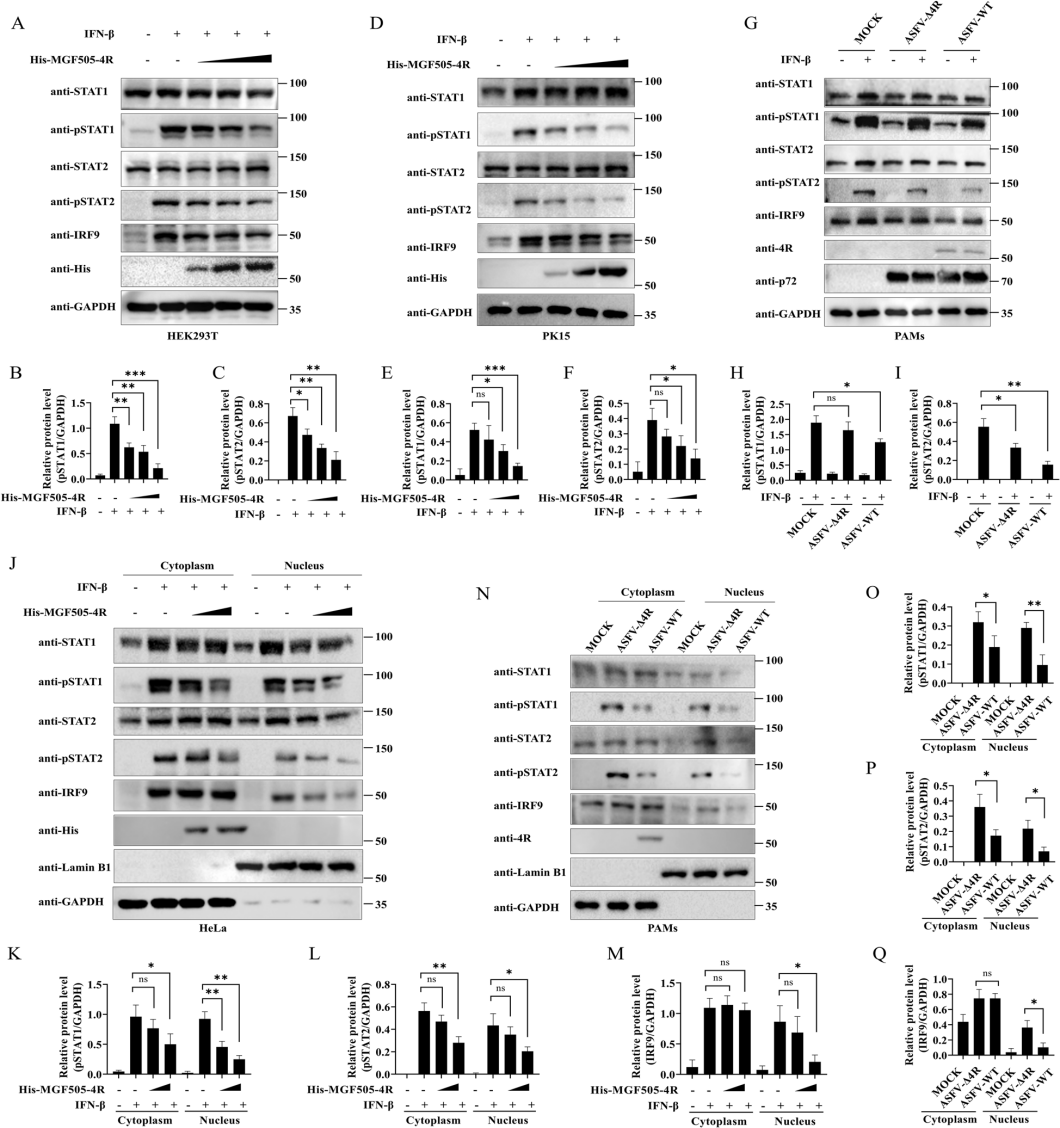
**ASFV MGF505-4R prevents the migration of phosphorylated STAT1, phosphorylated STAT2, and IRF9 into the nucleus.** **A**–**C** HEK293T cells were transfected with increasing doses of His-MGF505-4R plasmids for 24 h and then treated with IFN-β for another 2 h before western blot and ImageJ analysis. **D**–**F** PK15 cells were treated as in (**A**) and analyzed by western blot, followed by quantification using ImageJ. **G**–**I** PAMs were mock-infected or infected with ASFV-WT or ASFV-Δ4R for 36 h and then treated or untreated with IFN-β for an additional 2 h before western blot and ImageJ analysis. **J**–**M** HeLa cells were transfected with increasing doses of His-MGF505-4R for 24 h and then treated with IFN-β for an additional 2 h. Subsequently, the cytoplasm and nucleus were isolated and subjected to western blot and ImageJ analysis. **N**–**Q** PAMs were mock-infected or infected with ASFV-WT or ASFV-Δ4R for 36 h. Subsequently, the cytoplasm and nucleus were isolated and subjected to western blot and ImageJ analysis. *n* = 3; *p < 0.05; **p < 0.01; ***p < 0.001; ns, not significant.

**Figure 10:**
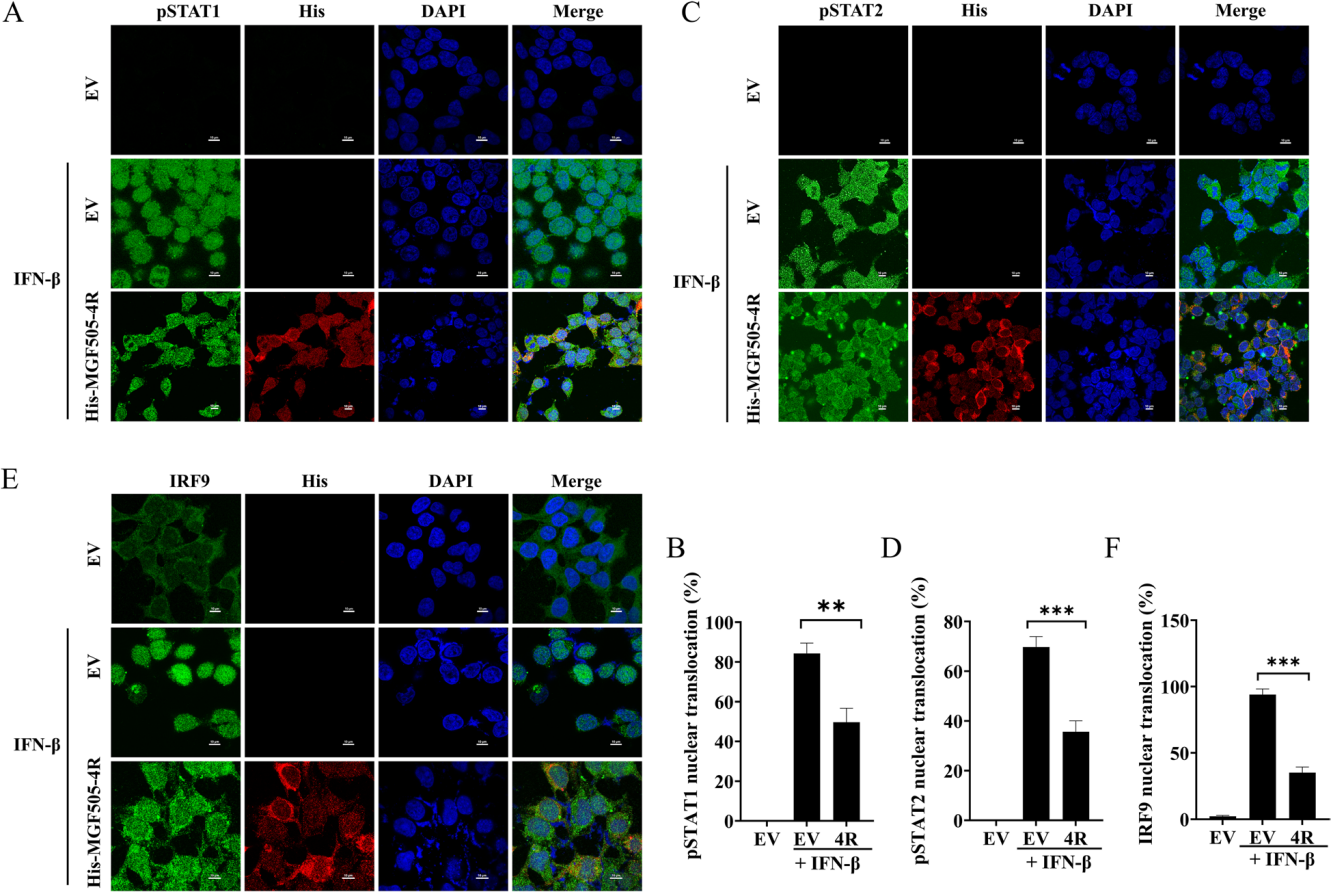
**ASFV MGF505-4R prevents the migration of phosphorylated STAT1, phosphorylated STAT2, and IRF9 into the nucleus.** **A**–**F** HEK293T cells transfected with either an empty vector (EV) or MGF505-4R plasmids were stimulated with or without IFN-β for 2 h. The nuclear translocation of phosphorylated STAT1 (pSTAT1), phosphorylated STAT2 (pSTAT2), and IRF9 was then analyzed by IFA using indicated antibodies and quantified with ImageJ. *n* = 3; **p < 0.01; ***p < 0.001.

We further investigated whether MGF505-4R interferes with the phosphorylation of STAT1 and STAT2, which is critical for the generation of the STAT1-STAT2 dimer and its subsequent binding to IRF9. HEK293T and PK15 cells overexpressing MGF505-4R were treated with IFN-β, western blot analysis revealed that MGF505-4R dose-dependently inhibited IFN-β-induced phosphorylation of both STAT1 and STAT2 in HEK293T (Figures 9A–C) and PK15 cells (Figures 9D-F), as quantified by ImageJ. Furthermore, compared with infection with ASFV-WT, infection with ASFV-Δ4R increased the phosphorylation of STAT1 and STAT2 following IFN-β treatment. However, it remained lower than the levels observed in the mock-infected group treated with IFN-β (Figures 9G–I). We further explored the effects of MGF505-4R on the nuclear translocation of ISGF3, nuclear-cytoplasmic fractionation experiments combined with ImageJ analysis demonstrated that MGF505-4R significantly inhibited IFN-β-induced nuclear translocation of ISGF3 components, including phosphorylated STAT1, phosphorylated STAT2, and IRF9 in HeLa cells (Figures 9J–M). Consistently, compared with infection with ASFV-WT, infection with ASFV-Δ4R increased nuclear translocations of phosphorylated STAT1, phosphorylated STAT2, and IRF9 in PAMs (Figures 9N–Q). To validate these findings, we performed IFA experiments and quantified the results using ImageJ software, the data revealed that MGF505-4R obviously blocked the IFN-β-induced nuclear translocation of phosphorylated STAT1 (Figures 10A and B), phosphorylated STAT2 (Figures 10C and D), and IRF9 (Figures 10E and F). These findings highlight the role of MGF505-4R in facilitating immune evasion during ASFV infection.

## Discussion

cGAS was initially recognized in 2013 as being responsible for initiating the innate immune response by catalyzing the synthesis of 2′3'-cGAMP [8]. As a cytosolic DNA sensor, cGAS is essential for immune defense against DNA-containing pathogens [29]. However, viruses have evolved diverse strategies to regulate the post-translational modifications (PTMs) of cGAS to interfere with its function, including but not limited to phosphorylation, ubiquitination, acetylation, SUMOylation, and methylation. These modifications subsequently affect its enzymatic activity, DNA binding capacity, and protein stability [30–32]. Ubiquitination is closely related to protein stability and commonly serves as a degradation signal recognized by autophagy receptors via ubiquitin-binding domains, which subsequently direct the degradation of proteins by autophagosomes [33–35]. For example, PCV2 promotes the K48-linked polyubiquitination of cGAS at lysine 389, which facilitates the binding of cGAS to p62, leading to the autophagic degradation of cGAS [36]. Another study revealed that nonstructural protein 2 of Porcine deltacoronavirus facilitates K48-linked ubiquitination of porcine STING for proteasomal degradation [37]. ASFV MGF300-2R protein facilitates the K27-linked polyubiquitination of IKKα and IKKβ, which is subsequently identified by TOLLIP for autophagy degradation [38]. Additionally, the PRV UL21 protein promotes the K27-linked ubiquitination of cGAS through recruiting E3C, thereby leading to TOLLIP-mediated autophagic degradation [39]. However, in our study, we found that MGF505-4R did not significantly affect the ubiquitination of cGAS (Additional file 6). Notably, TOLLIP reportedly still interacts with unmodified cargo when its ubiquitin-binding domain is mutated. This finding suggests that the ubiquitination signal may enhance substrate degradation but does not play an indispensable role [40]. Another mechanism by which TOLLIP recognizes and directs substrates for degradation in the autophagosome may exist and requires further investigation. Most recently, it was reported that MGF505-4R likely interacts with TRAF3 to interfere with the IFN-α/β pathway. TRAF3 is recruited upon the activation of MAVS, indicating that MGF505-4R may also be involved in the RIG-I-MAVS pathway to regulate immune responses [27].

The IFN-mediated pathway plays an indispensable role in combatting pathogen invasion [41]. Many viral proteins impede the activation of STAT1 and STAT2 to evade immune defense. For example, ASFV pI7L engages with STAT1, thereby impeding its phosphorylation and homodimerization [42]. ASFV MGF505-7R interacts with IRF9, thereby inhibiting the formation of ISGF3 heterotrimers [43]. ASFV MGF360-12L blocks the ISRE promoter activation by reducing total protein levels of IRF9 [44]. In our study, we found that MGF505-4R effectively obstructed the IFN-β signaling pathway through its interaction with STAT1 and STAT2. Specifically, MGF505-4R inhibited the phosphorylation of both STAT1 and STAT2, resulting in the suppression of ISGF3 formation and its subsequent translocation to the nucleus. Interestingly, Dupré's study indicated that both MGF360-12L and MGF505-4R exhibit no antagonistic effects on IFN-β-induced ISRE promoter activation [27]. This may be due to the different experimental conditions, including transfection duration, as well as the timing and dosage of IFN-β treatment.

To assess the antagonistic effects of MGF505-4R on innate immune responses during ASFV infection, we constructed an MGF505-4R-deficient ASFV mutant via homologous recombination. Compared with the ASFV-WT strain, the ASFV-Δ4R mutant increased the secretion of IFN-β and increased the mRNA expression of antiviral genes in PAMs (Figures 1E and F), which is consistent with findings related to MGF505-4R overexpression. Additionally, ASFV-Δ4R partially lost its inhibitory effects on both the mRNA expression of ISGs and the phosphorylation of STAT1 and STAT2 (Figures 6C and 9G–I). However, compared with mock infection, ASFV-Δ4R infection still significantly restrained the activation of the IFN-β-mediated pathway. This effect is likely attributed to the intricate immune evasion strategies conferred by various ASFV-encoded proteins [42–47]. This may explain why the deletion of MGF505-4R did not significantly impact the growth characteristics of ASFV, although the TCID_50_ of ASFV-WT at 48 hpi and 72 hpi was slightly greater than that of ASFV-Δ4R (Figure 1D). Notably, the L11L gene does not influence viral replication in PAMs but significantly attenuates the pathogenicity of the ASFV SY18 strain in vivo [48]. In another study, the I7L gene deletion mutant presented growth kinetics similar to those of its parental strain, whereas it presented diminished virulence in porcine subjects [42]. Therefore, whether the deletion of the MGF505-4R gene from ASFV-SXH1 impacts viral pathogenicity requires further investigation in vivo.

In our study, we demonstrated interactions between MGF505-4R and multiple host proteins (cGAS, TOLLIP, STAT1, and STAT2) using co-IP and colocalization analysis. Notably, MGF505-4R significantly enhances the association between cGAS and TOLLIP, suggesting that MGF505-4R may function as a scaffold to stabilize or facilitate their interaction. Interestingly, we observed a basal-level interaction between cGAS and TOLLIP even in the absence of MGF505-4R, indicating that these proteins may also bind directly. However, whether the interactions among cGAS, TOLLIP, and MGF505-4R are direct or indirect, as well as the precise binding sites, requires further investigation. In addition, we demonstrated that MGF505-4R interacts with both STAT1 and STAT2, suppressing phosphorylation at Tyr701 (STAT1) and Tyr690 (STAT2), leading to the inhibition of STAT1-STAT2-IRF9 (ISGF3) complex formation. Structural studies suggest that the SH2 domain of STAT1/STAT2 mediates their heterodimerization via phospho-Tyr701 (STAT1) and phospho-Tyr690 (STAT2). Additionally, the coiled-coil domain (CCD) of STAT2 is critical for recruiting IRF9 to form the functional ISGF3 trimer [49]. We hypothesize that MGF505-4R may competitively bind to the SH2 or CCD of STAT1 and STAT2, leading to decreased interactions among STAT1, STAT2 and IRF9. Future experiments should precisely map these binding sites to validate this mechanism.

**Figure 11:**
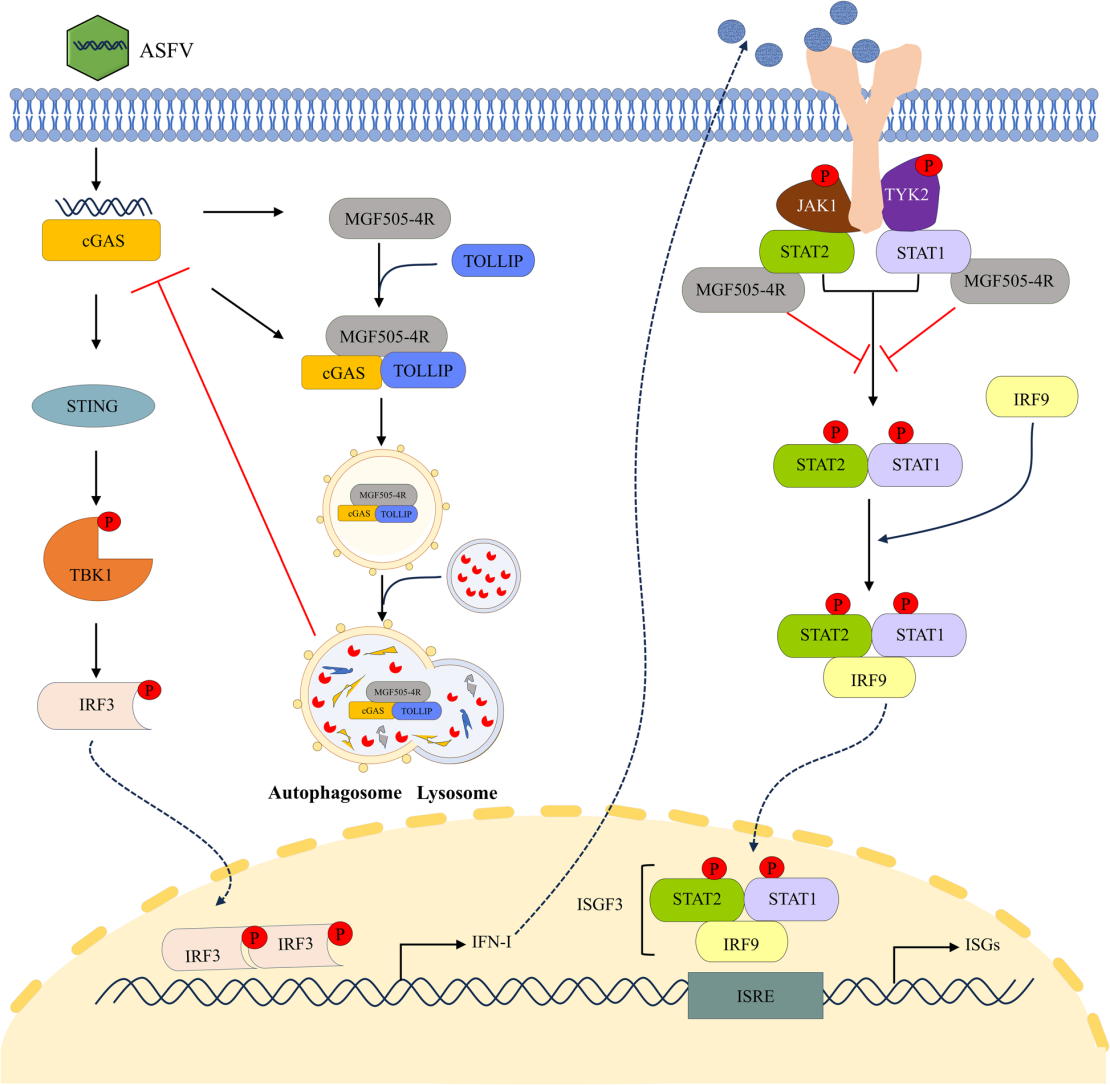
**Schematic diagram illustrating the antagonistic mechanism employed by ASFV MGF505-4R on IFN-I responses**. MGF505-4R downregulates the protein level of cGAS by triggering TOLLIP-mediated selective autophagy, thereby antagonizing the IFN-I production induced by the cGAS‒STING pathway. Additionally, MGF505-4R interacts with STAT1 and STAT2, which subsequently interferes with the formation of the ISGF3 heterotrimer and its nuclear translocation, thereby reducing the production of ISGs.

This study identified a novel strategy employed by ASFV MGF505-4R to antagonize immune defense by interfering with both the cGAS‒STING pathway and the JAK‒STAT pathway. This finding enhances our understanding of the immune evasion mechanisms of ASFV and provides valuable insights for antiviral research against ASFV (Figure 11).

## Supplementary Information

**Additional file 1. Primers used for ASFV-WT and ASFV-Δ4R detection**.

**Additional file 2. Primers used for qRT-PCR in this study.**

**Additional file 3. The sequence of siRNA used in this study.**

**Additional file 4. ASFV MGF505-4R potentiates PRV-GFP replication.** (A and B) HEK293T cells were transfected with increasing doses of His-MGF505-4R plasmid, along with pGL3-IFN-β-Luc and pRL-TK, for 24 h and then infected with 0.1 MOI of PRV-GFP for 24 h before luciferase assays, western blot detection and fluorescence microscope observation. (C and D) SK6 cells were treated in the same manner as described for HEK293T cells for luciferase assays, western blot detection, and fluorescence microscope observation. *n* = 3; *, *p* < 0.05; **, *p* < 0.01; ***, *p* < 0.001; ns, not significant.

**Additional file 5. Validation of siRNA knockdown effects.** (A) HeLa cells were transfected with either negative control siRNA (siNC) or siRNA targeting cGAS (sicGAS) for 24 h before western blot analysis. (B) HeLa cells were transfected with siNC or siRNA targeting ATG5 (siATG5) for 24 h before western blot analysis. (C) HeLa cells were transfected with siNC or siRNA targeting TOLLIP (siTOLLIP) for 24 h before western blot analysis.

**Additional file 6. MGF505-4R does not affect the polyubiquitination of cGAS.** HEK-293 T cells were co-transfected with His-MGF505-4R, Flag-cGAS, and HA-Ubi plasmids for 24 h. Subsequently, the cells were treated with NH4Cl, CQ or MG132 for 12 h before western blot and co-IP detection.

## Data Availability

All data generated or analysed during this study are included in this published article [and its supplementary information files].
