# Supplementary material for: African swine fever virus MGF505-4R facilitates cGAS degradation through TOLLIP-mediated selective autophagy and inhibits the formation of ISGF3 to evade innate immunity
Source: Vet Res. 2025 Jul 5;56:137. doi: 10.1186/s13567-025-01569-x (PMC12228400; doi:10.1186/s13567-025-01569-x)
Supplement: Supplementary file 1 — Additional file 1. Primers used for ASFV-WT and ASFV-Δ4R detection. [file 13567_2025_1569_MOESM1_ESM.docx]

**Additional file 1 Primers used for ASFV-WT and ASFV-Δ4R detection.**

| Primers | Sequence (5’ to 3’) |
| --- | --- |
| MGF505-4R Center-forward  MGF505-4R Center-reverse  B646L ORF-forward  B646L ORF-reverse  MGF505-4R ORF-forward  MGF505-4R ORF-reverse | GACACATGAGGGAGAGAATCA  35963-35984  CGTTTTTATGGATGCACAATTGG  36416-36439  ATGGCATCAGGAGGAGCTTTT  105158-105179  TTAGGTACTGTAACGCAGCAC  103217-103238  ATGTTTTCTCTTCAAGACATCTGT  35501-35525  TTACTCAGATTTCGATAGAATTTCT  37022-37047 |
