# Supplementary material for: African swine fever virus MGF505-4R facilitates cGAS degradation through TOLLIP-mediated selective autophagy and inhibits the formation of ISGF3 to evade innate immunity
Source: Vet Res. 2025 Jul 5;56:137. doi: 10.1186/s13567-025-01569-x (PMC12228400; doi:10.1186/s13567-025-01569-x)
Supplement: Supplementary file 2 — Additional file 2. Primers used for qRT-PCR in this study. [file 13567_2025_1569_MOESM2_ESM.docx]

**Additional file 2** **Primers used for qRT-PCR in this study.**

| Primers | Sequence (5’ to 3’) |
| --- | --- |
| Human IFN-β-forward  Human IFN-β-reverse  Human ISG54-forward  Human ISG54-reverse  Human ISG56-forward  Human ISG56-reverse  Human ISG15-forward  Human ISG15-reverse  Human MX1-forward  Human MX1-reverse  Human GAPDH-forward  Human GAPDH-reverse  Pig IFN-β-forward  Pig IFN-β-reverse  Pig ISG54-forward  Pig ISG54-reverse  Pig ISG56-forward  Pig ISG56-reverse  Pig ISG15-forward  Pig ISG15-reverse  Pig MX1-forward  Pig MX1-reverse  Pig GAPDH-forward  Pig GAPDH-reverse | TCTTTCCATGAGCTACAACTTGCT  GCAGTATTCAAGCCTCCCATTC  AATAGGACACGCTGTGGCTC GGTGGATGGCCTTGTCTTCA  GAAGGATGGGCCTTGCTGAA  CAGGCGATAGGCAGAGATCG  GGTGCAAAGCTTCAGAGACC  GTCAGCCAGACCTCATAGGC  CAGGACATTTGAGACAATCGTG  TCGAAACATCTGTGAAAGCAAG  AAATTCCATGGCACCGTCAA  TGGTTCACACCCATGACGAA  CACTGGCTGGAATGAAACCG  AATGGTCATGTCTCCCCTGG  CTGGCAAAGAGCCCTAAGGA  CTCAGAGGGTCAATGGAATTCC  TTAGAAAACAGGGTCTTGGAGGAG  CGTAAGGTAATACAGCCAGGCATA  GATCGGTGTGCCTGCCTTC  CGTTGCTGCGACCCTTGT  AGCGCAGTGACACCAGCGAC  GCCCGGTTCAGCCTGGGAAC  ACATGGCCTCCAAGGAGTAAGA  GATCGAGTTGGGGCTGTGACT |
