# Supplementary material for: African swine fever virus MGF505-4R facilitates cGAS degradation through TOLLIP-mediated selective autophagy and inhibits the formation of ISGF3 to evade innate immunity
Source: Vet Res. 2025 Jul 5;56:137. doi: 10.1186/s13567-025-01569-x (PMC12228400; doi:10.1186/s13567-025-01569-x)
Supplement: Supplementary file 3 — Additional file 3. The sequence of siRNA used in this study. [file 13567_2025_1569_MOESM3_ESM.docx]

**Additional file 3 The sequence of siRNA used in this study**

| Oligo Name | Sequence (5’ to 3’) |
| --- | --- |
| siRNA-cGAS 1# sense  siRNA-cGAS 1# antisense  siRNA-cGAS 2# sense  siRNA-cGAS 2# antisense  siRNA-cGAS 3# sense  siRNA-cGAS 3# antisense  siRNA-ATG5 1# sense  siRNA-ATG5 1# antisense  siRNA-ATG5 2# sense  siRNA-ATG5 2# antisense  siRNA-ATG5 3# sense  siRNA-ATG5 3# antisense  siRNA-TOLLIP 1# sense  siRNA-TOLLIP 1# antisense  siRNA-TOLLIP 2# sense  siRNA-TOLLIP 2# antisense  siRNA-TOLLIP 3# sense  siRNA-TOLLIP 3# antisense | ccu gcu gua aca cuu cuu a/dt//dt//dt//dt/  uaa gaa gug uua cag cag g/dt//dt//dt//dt/  cgu gaa gau uuc ugc acc u/dt//dt//dt//dt/  agg ugc aga aau cuu cac g/dt//dt//dt//dt/  cuu uga uaa cug cgu gac a/dt//dt//dt//dt/  ugu cac gca guu auc aaa g/dt//dt//dt//dt/  gaa ggu uau gag aca aga a/dt//dt/  uuc uug ucu cau aac cuu c/dt//dt/  gaa aga agc uga ugc uuu a/dt//dt/  uaa agc auc agc uuc uuu c/dt//dt/  gcu aua uca gga uga gau a/dt//dt/  uau cuc auc cug aua uag c/dt//dt/  cga gau cuu cga uga gag a/dt//dt/  ucu cuc auc gaa gau cuc g/dt//dt/  agg ugg agg aca agu ggu a/dt//dt/  uac cac uug ucc ucc acc u/dt//dt/  gcu gga aua agg uca ucc a/dt//dt/  ugg aug acc uua uuc cag c/dt//dt/ |
