# Supplementary material for: African swine fever virus MGF505-4R facilitates cGAS degradation through TOLLIP-mediated selective autophagy and inhibits the formation of ISGF3 to evade innate immunity
Source: Vet Res. 2025 Jul 5;56:137. doi: 10.1186/s13567-025-01569-x (PMC12228400; doi:10.1186/s13567-025-01569-x)
Supplement: Supplementary file 4 — Additional file 4. ASFV MGF505-4R potentiates PRV-GFP replication. (A and B) HEK293T cells were transfected with increasing doses of His-MGF505-4R plasmid, along with pGL3-IFN-β-Luc and pRL-TK, for 24 h and then infected with 0.1 MOI of PRV-GFP for 24 h before luciferase assays, western blot detection and fluorescence microscope observation. (C and D) SK6 cells were treated in the same manner as described for HEK293T cells for luciferase assays, western blot detection, and fluorescence microscope observation. n = 3; *, p < 0.05; **, p < 0.01; ***, p < 0.001; ns, not significant. [file 13567_2025_1569_MOESM4_ESM.docx]

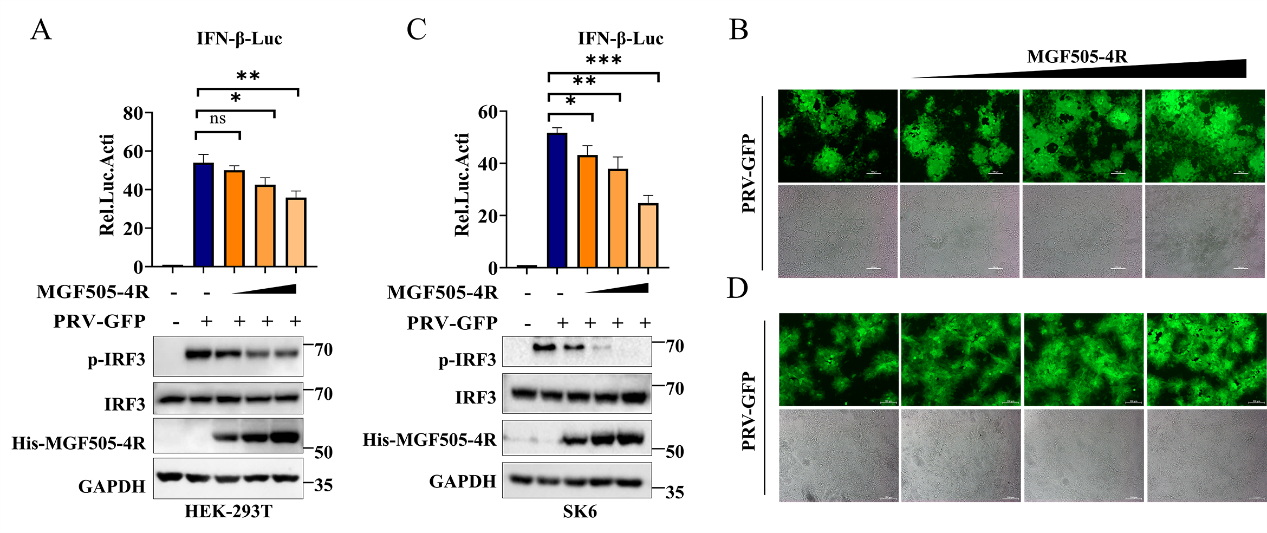


**Additional file 4** **ASFV MGF505-4R potentiates PRV-GFP replication**. (A and B) HEK293T cells were transfected with increasing doses of His-MGF505-4R plasmid, along with pGL3-IFN-β-Luc and pRL-TK, for 24 h and then infected with 0.1 MOI of PRV-GFP for 24 h before luciferase assays, western blot detection and fluorescence microscope observation. (C and D) SK6 cells were treated in the same manner as described for HEK293T cells for luciferase assays, western blot detection, and fluorescence microscope observation. *n* = 3; *, *p* < 0.05; **, *p* < 0.01; ***, *p* < 0.001; ns, not significant.
