# Supplementary material for: African swine fever virus MGF505-4R facilitates cGAS degradation through TOLLIP-mediated selective autophagy and inhibits the formation of ISGF3 to evade innate immunity
Source: Vet Res. 2025 Jul 5;56:137. doi: 10.1186/s13567-025-01569-x (PMC12228400; doi:10.1186/s13567-025-01569-x)
Supplement: Supplementary file 5 — Additional file 5. Validation of siRNA knockdown effects. (A) HeLa cells were transfected with either negative control siRNA (siNC) or siRNA targeting cGAS (sicGAS) for 24 h before western blot analysis. (B) HeLa cells were transfected with siNC or siRNA targeting ATG5 (siATG5) for 24 h before western blot analysis. (C) HeLa cells were transfected with siNC or siRNA targeting TOLLIP (siTOLLIP) for 24 h before western blot analysis. [file 13567_2025_1569_MOESM5_ESM.docx]

**
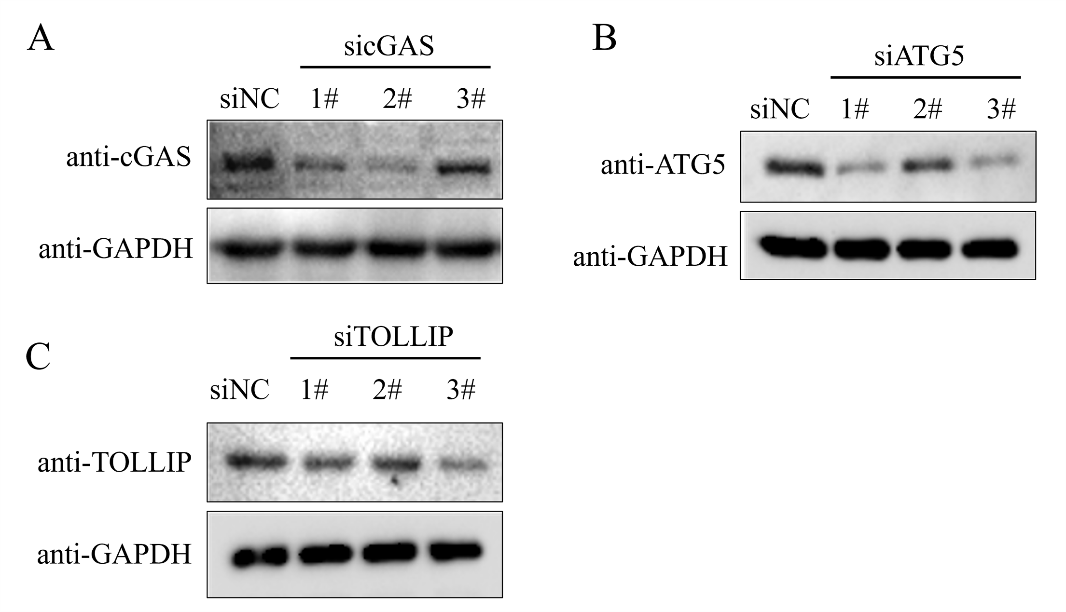
**

**Additional file 5 Validation of siRNA knockdown effects.** (A) HeLa cells were transfected with either negative control siRNA (siNC) or siRNA targeting cGAS (sicGAS) for 24 h before western blot analysis. (B) HeLa cells were transfected with siNC or siRNA targeting ATG5 (siATG5) for 24 h before western blot analysis (C) HeLa cells were transfected with siNC or siRNA targeting TOLLIP (siTOLLIP) for 24 h before western blot analysis.
