# Supplementary material for: African swine fever virus MGF505-4R facilitates cGAS degradation through TOLLIP-mediated selective autophagy and inhibits the formation of ISGF3 to evade innate immunity
Source: Vet Res. 2025 Jul 5;56:137. doi: 10.1186/s13567-025-01569-x (PMC12228400; doi:10.1186/s13567-025-01569-x)
Supplement: Supplementary file 6 — Additional file 6. MGF505-4R does not affect the polyubiquitination of cGAS. HEK-293 T cells were co-transfected with His-MGF505-4R, Flag-cGAS, and HA-Ubi plasmids for 24 h. Subsequently, the cells were treated with NH4Cl, CQ or MG132 for 12 h before western blot and co-IP detection. [file 13567_2025_1569_MOESM6_ESM.docx]

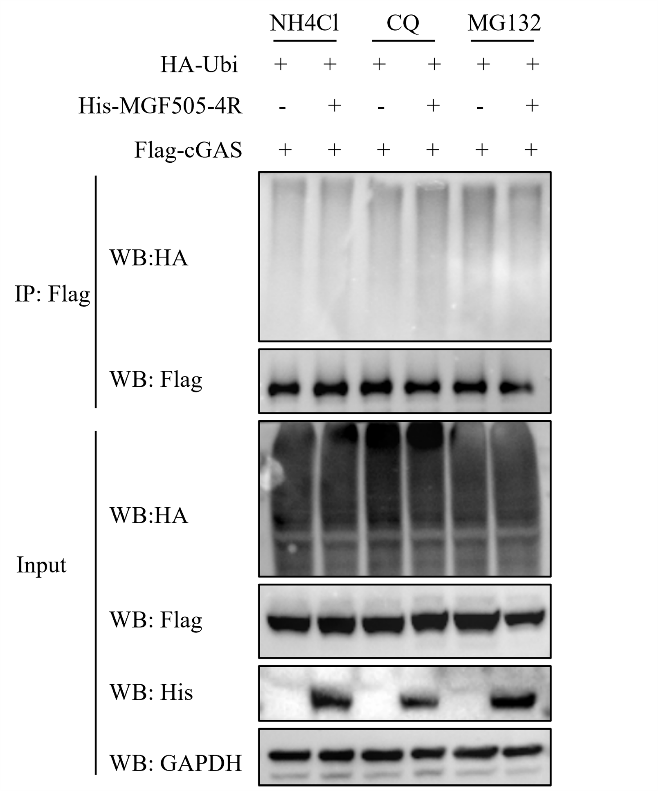


**Additional file 6 MGF505-4R does not affect the polyubiquitination of cGAS.** HEK-293T cells were co-transfected with His-MGF505-4R, Flag-cGAS, and HA-Ubi plasmids for 24 h. Subsequently, the cells were treated with NH4Cl, CQ or MG132 for 12 h before western blot and co-IP detection.
